# Double-edged effects of aquatic environmental biofilms on *Batrachochytrium dendrobatidis* growth and inhibition

**DOI:** 10.1093/ismeco/ycaf185

**Published:** 2025-10-16

**Authors:** Renwei Chen, Caitlin L Nordheim-Maestas, Cheryl J Briggs

**Affiliations:** Marine Science Institute, University of California, Santa Barbara, CA 93106, USA; Ecology, Evolution, and Marine Biology, University of California, Santa Barbara, CA 93106, USA; Marine Science Institute, University of California, Santa Barbara, CA 93106, USA; Ecology, Evolution, and Marine Biology, University of California, Santa Barbara, CA 93106, USA

**Keywords:** chytrid fungus, *Batrachochytrium dendrobatidis*, aquatic microbiota, biofilms, planktonic microorganisms, fungal-microbiome interactions

## Abstract

*Batrachochytrium dendrobatidis* (Bd) is an aquatic chytrid fungus that infects amphibians and has the potential to remain viable outside of hosts. However, the role of aquatic microbiota in influencing Bd growth and survival remains insufficiently understood. In this study, we demonstrated that in the absence of amphibian hosts, aquatic environmental (AE) biofilms supported the development of Bd, allowing it to complete its life cycle for a short period; whereas aquatic planktonic microorganisms did not. However, exposure of Bd zoospores to AE biofilms or planktonic microorganisms resulted in a significant reduction in Bd DNA within a week. These results suggest a dual role of aquatic biofilms in both supporting Bd growth and inhibiting it simultaneously. Moreover, Bd monolayers, composed mainly of zoosporangia, rapidly declined when exposed to AE planktonic microorganisms. Laboratory-formulated nutrients further enhanced the Bd-inhibitory effect of AE microbiota, suggesting that competition for shared nutrients plays a role in this interaction. This study advances our understanding of the complex interactions between Bd and aquatic microbial communities, underscores the ecological significance of biofilm-associated environments, and supports the potential of microbiota-informed interventions for controlling chytridiomycosis in amphibians.

## Introduction

The chytrid fungus *Batrachochytrium dendrobatidis* (Bd) infects amphibians and can cause chytridiomycosis in susceptible species. Bd has a wide host range and can infect many amphibian species, although not all individuals are susceptible. For example, Olson *et al.* reported that 1375 out of 2525 individuals tested (55%) were infected [1]. The infection outcomes vary greatly, ranging from asymptomatic carriage in some species to lethal chytridiomycosis in others [2]. This disease has been implicated in amphibian population declines and extinctions worldwide [3–5]. Since its discovery in 1999 [6], considerable theoretical and empirical research has focused on Bd’s ability to persist and potentially replicate outside its amphibian hosts [7–9]. Environmental reservoirs that decouple pathogen abundance from host dynamics may allow Bd to survive during periods of scarcity [10, 11], which in turn contribute to its long-term persistence and possibly influence host population dynamics [3].

Bd has a biphasic life cycle consisting of a motile, infective zoospore stage (2–5 *μ*m) and a sessile, reproductive zoosporangium stage (10–40 *μ*m) [12]. A dormant spore stage has not been identified. Bd can persist in the environment independent of amphibian hosts: It survives for days to weeks in water, moist soil, and on abiotic surfaces [13, 14]. It can also be cultured indefinitely on simple laboratory media such as tryptone-gelatin-hydrolysate-lactose or tryptone broth (TB) [15–17], suggesting it may utilize non-amphibian substrates in nature. Under sterile conditions, Bd has been shown to persist for up to 7 weeks in lake water [13], 3 months in moist river sand [18], and to grow on various keratinized substances [19]. However, successful growth of Bd on abiotic substrates has been observed almost exclusively in sterile environments, as Bd is readily overgrown under non-sterile conditions [18, 20]. In natural habitats, Bd DNA has been detected on riparian vegetation where amphibians recently rested [14], as well as in water and sediment [21]. Beyond amphibians, Bd has also been associated with or shown to infect a wide range of non-amphibian organisms, including nematodes [22, 23], crustaceans [24–26], midges [27], reptiles [28, 29], birds [30–32], and zebrafish [33]. These findings raise the possibility of alternative hosts or passive carriers contributing to Bd persistence and transmission in complex ecosystems.

Bd transmission occurs both through direct contact between infected and uninfected amphibians [34], and indirectly via environmental sources such as water bodies or contaminated substrates, where motile zoospore released from infected amphibians can encounter and infect susceptible hosts [35]. Aquatic environments harbor diverse microbial assemblages that can influence Bd growth and pathogenicity [36]. Certain amphibian skin symbiotic bacteria exhibit inhibitory functions that suppress the proliferation and virulence of Bd, offering a natural form of biological control [37–41]. Multiple mechanisms underlie this inhibitory effect. For instance, amphibian skin glands secrete antimicrobial peptides that play a role in defense against fungal pathogens [42]. The activity of these peptides can be potentiated by probiotic bacteria, which enhance their antifungal properties and promote the growth of Bd-inhibiting bacterial taxa [43, 44]. Bacteria produce antifungal compounds such as violacein, prodigiosin, and short-chain organic acids that disrupt Bd membrane integrity, cellular respiration, or reproduction [45]. Additionally, microbial interactions with environmental factors, such as temperature, humidity, or other microbes, can indirectly inhibit Bd [46–52].

Increasingly, studies have begun to investigate the influence of aquatic biofilms on Bd growth, driven by their distinct functional and physical properties inherent to biofilms [53, 54]. Biofilms are complex, surface-associated communities of microorganisms embedded in an extracellular matrix. This matrix, primarily composed of extracellular polymeric substances, including polysaccharides, proteins, lipids, and extracellular DNA, plays a crucial role in the biofilm’s structural integrity and function [55, 56]. Biofilm-forming microbes produce specific surface proteins, such as adhesins, which enable them to attach to both biotic and abiotic surfaces [57, 58]. These surface proteins also facilitate the recruitment and retention of other microorganisms within the biofilm, promoting the formation of multi-species communities [55, 56, 59]. Recent studies have shown that biofilms in aquatic environments exhibit Bd-inhibitory functions [41, 53, 54]. The study by Sentenac *et al.* reported that biofilms significantly decrease Bd survival, with the impact on zoospores varying based on biofilm composition under controlled conditions [54]. In that study, natural variations in biofilm composition were correlated with differential impacts of chytridiomycosis on amphibian populations, underscoring the ecological relevance of biofilm-pathogen interactions. Similarly, Chen *et al.* [53] demonstrated that both the thickness and richness of Bd-inhibitory bacteria within biofilms play complementary roles in preventing Bd establishment. Thick biofilms act as physical barriers to Bd colonization, while biofilms enriched with diverse inhibitory bacteria further suppress Bd [53]. In addition, numerous lactic acid bacteria found on amphibian skin have demonstrated strong adhesion and colonization capabilities, including hydrophilicity and biofilm formation. These biofilms may inhibit Bd via adhesion mechanisms involving electrostatic and hydrophobic interactions [60]. Each of these mechanisms may contribute to protecting amphibians from Bd and highlight promising avenues for the development of potential biological control strategies targeting this fungus.

Notably, Bd itself is capable of forming monolayers that resemble single-species biofilms, when cultured in both nutrient-rich liquid medium and nutrient-poor natural pond and lake water [61]. In this study, Silva *et al.* observed that single-species Bd biofilms exhibited enhanced resistance to environmental stressors, such as pH fluctuations and chemical exposure; however, they were less resistant to elevated temperatures compared to planktonic Bd. This observation raises an intriguing question that we will begin to explore here: might Bd monolayers gain protection from interactions with aquatic environmental (AE) microorganisms, rather than being solely inhibited by them?

Understanding the interactions between Bd and aquatic microbial communities is important for developing effective strategies to mitigate the impact of chytridiomycosis on amphibian populations. However, the physical interactions between Bd and AE biofilms remain poorly understood. In this study, we examined how AE biofilms influence Bd zoospore attachment, life cycle progression, and growth inhibition. We also assessed the role of AE planktonic microorganisms in modulating Bd zoospores and zoosporangia when they form surface-attached monolayers, with particular attention to potential nutritional competition between Bd and surrounding microbial communities. Notably, we identified a novel dual effect of AE biofilms: they allowed Bd’s life cycle completion for a short period while simultaneously inhibiting its proliferation. These findings deepen our understanding of the complex interactions shaping amphibian disease dynamics and ecosystem ecology.

## Materials and methods

### Field sites

To investigate the influence of environmental microbiota on Bd growth, we conducted experiments using samples from two distinct regions in California. The region that we will refer to as SBNCOS is a small, single-pond site in the North Campus Open Space near the University of California Santa Barbara campus. The location of this site near our institution allowed for repeated access and supported a broader range of experiments across multiple media types. In contrast, the region that we will refer to as SFEB (San Francisco East Bay) spans a large geographic area and consists of a subset of the ponds that have been extensively studied for amphibians and their pathogens and parasites as part of a larger research program [62–66]. The SFEB sites sampled for AE microbes and/or biofilms in this study include up to 15 ponds across three parks or reserves, including the University of California Natural Reserve System’s Blue Oak Ranch Reserve (BORR), the East Bay Regional Park’s Garin/Dry Creek Regional Park (GD), and Pleasanton Ridge Regional Park (PR). These geographic and logistical differences resulted in distinct experimental designs and justified treating the two regions separately in our analyses and interpretations. All pond sites are shown on a georeferenced map with latitude and longitude coordinates (Fig. S1). Sampling in the SFEB region took place between March and May 2022, during which water temperature was between 18°C and 25°C. SBNCOS samples were collected in May 2021, August 2023, and April 2024. Amphibians were present at all ponds during the study period, and Bd was detected by PCR in 25% of sampled individuals (*n* = 40) in SFEB in May 2022, and 17% (1 of 6) in the SBNCOS pond in February 2021 (Table S1).

### AE microbiota sampling

AE biofilms were collected from a pond in SBNCOS, and from nine ponds in the SFEB region, specifically BORR ponds CABIN, GRAMPS, and WEST, GD ponds GDPND005, GDPND006, and GDPND009, and PR ponds PRPND004, PRPND009 and PRPND010. The procedure of AE biofilm collection involved submerging a nylon mesh pouch housing a 12-well tissue culture plate (Fisherbrand™, Cat. No, FB012928, Thermo Fisher Scientific, MA) and a Superfrost™ Plus microscopic slide (Fisherbrand™, Cat. No. 12-550-15, Fisher Scientific, PA) within the pond environment for 1 week. A photograph of *in situ* sample collecting device is provided in Fig. S2 to illustrate the setup. Subsequently, the devices were carefully retrieved and transported to the laboratory while maintaining their submersion within an ample volume of pond water sourced from the respective collection pond.

Pond water with its indigenous AE microorganisms was collected into clean plastic bottles from the same ponds described above, and from six additional ponds in the SFEB region, namely BORR ponds BARN and NORTH, GD ponds GDPND004 and GDPND008, and PR ponds PRPND002 and PRPND003. Pond water remained at room temperature overnight, and was filtered the next day on Day 0 of the experiment.

### Experiments

#### Experiment 1: Analysis of Bd life cycle in presence of AE planktonic microorganisms or biofilms

Microscopy was used to determine whether Bd can complete its life cycle in the presence of AE planktonic microorganisms or biofilms. Three labeling techniques were employed: (i) Zoospores were first labeled with a green fluorescent marker, i.e. retained during development but not passed to subsequent generations. These labeled zoospores were then exposed to either AE biofilms or pond water containing indigenous planktonic microorganisms. (ii) Concanavalin A (Con A) detects the presence of biofilms. (iii) Immunofluorescent staining with an anti-Bd antibody caused existing Bd zoospores and zoosporangia present at the time of staining to fluoresce green.

Bd zoospores from cultures (see Culture of Bd, below) were labeled with 2 *μ*m of green fluorescent-labeled (FL) Boron-dipyrromethene (BODIPY® 4,4-difluoro-3a,4adiaza-s-indacene, BODIPY™ 493/503, Cat. No. D3922, Molecular Probes, Inc. OR) for 90 min, followed by washing in sterile Milli-Q (MQ) water for three times to remove any excessive dye. Approximately 3 million FL-labeled Bd zoospores were exposed to the AE biofilms derived from SBNCOS on a microscopic slide submerged in 20 ml of microorganism-depleted pond water. Each treatment included three independent biological replicates, corresponding to sample taken on different dates, with one technical replicate per sample. The same amount of FL-labeled Bd zoospores were added to 20 ml of microorganism-retaining pond water in a 100 mm Petri dish containing a clean microscopic slide. Specimens were washed three times with phosphate-buffered saline and fixed in ice-cold methanol for 10 min on Days 1, 3, and 7. Fluorescent Alexa 594-conjugated Con A (Life Technologies) was used for the detection of the presence and distribution of the glycoconjugates (glycoproteins and glycolipids) on the cell surface or intracellular structures rich in the carbohydrates. Con A has been widely used to stain the extracellular polysaccharides in biofilms which are composed of a matrix of polysaccharides, proteins, and nucleic acids [67]. To distinguish between the initially introduced zoospores and newly released zoospores produced by zoosporangia that had developed within the biofilms during the 7-day exposure, we performed immunofluorescence staining at the end of the exposure period. Zoospores were not pre-labeled with BODIPY® dye to avoid potential interference with antibody binding. *Batrachochytrium* mouse monoclonal antibody, clone 5C4 (ISCA Diagnostics Limited, Exeter, UK) served as a primary antibody, followed by an Alexa Fluor 488-conjugated goat anti-mouse IgG (Life Technology) as a secondary antibody. The specimens were then mounted with aqueous anti-fade fluorescence mounting medium (Life Technology) and analyzed using a Leica DM6000 fluorescent microscope (Leica Microsystems, Wetzlar, Germany).

#### Experiment 2: Assessment of AE planktonic microorganisms’ inhibitory effects on Bd growth

**Figure 1:**
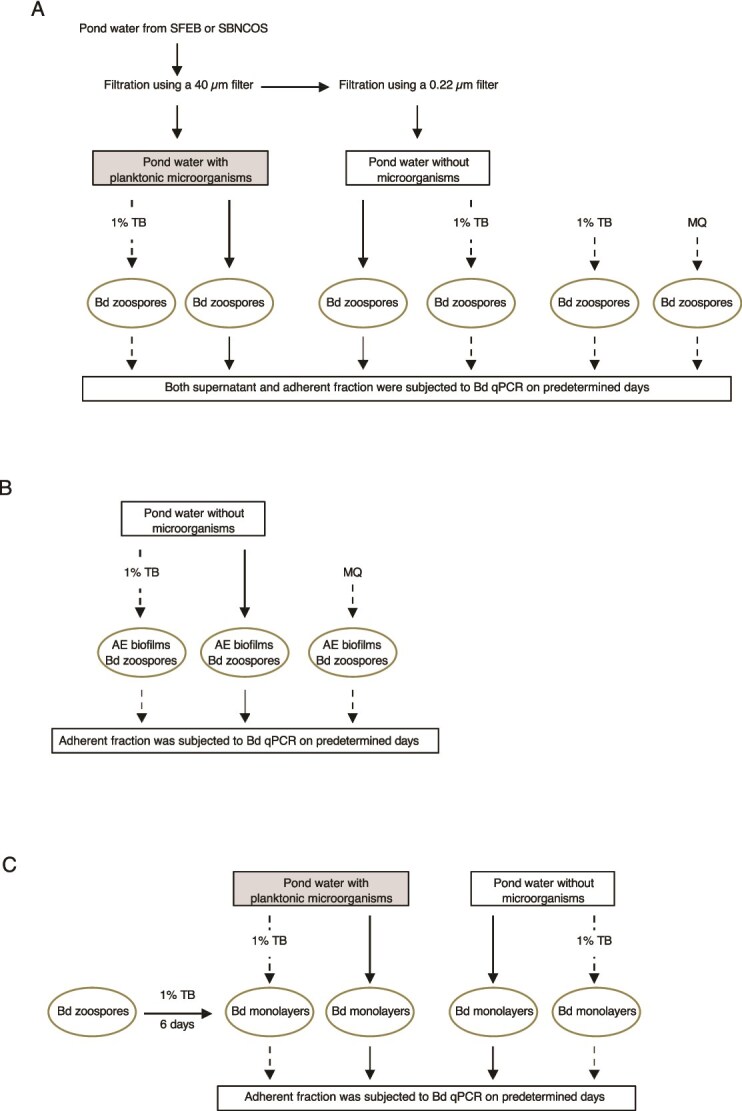
Diagram of the experimental design to assess the Bd-inhibitory effects of AE microbiota. Pond water collected from SFEB and SBNCOS was filtered using a 40 *μ*m filter and subsequently a 0.22 *μ*m syringe filter. (A) Approximately 1 million Bd zoospores were exposed to pond water collected from SFEB and SBNCOS, either retaining or depleted indigenous planktonic microorganisms. The SFEB dataset comprises 15 independent biological replicates (*n* = 15), with each sample collected from a different pond, in contrast to the SBNCOS dataset, which consists of three technical replicates from a single pond (*n* = 3). (B) Bd zoospores were exposed to AE biofilms in microorganism-depleted pond water collected from SFEB (*n* = 9) and SBNCOS (*n* = 3). (C) Bd zoospores were initially cultured in 1% TB in 12-well plates for 6 days to establish monolayers, and then exposed to the pond water collected from SFEB (*n* = 9) and SBNCOS (*n* = 3), with or without AE planktonic microorganisms. For SBNCOS samples, parallel treatments included 1% TB in all Experiments A, B, and C, and sterile MQ water in Experiments A and B. Solid arrows indicate AE microbiota derived from 15 ponds in SFEB and one pond in SBNCOS, while dashed arrows denote samples exclusively from the SBNCOS pond. Bd-exposed samples were collected on Days 1 and 7 for SFEB, and on Days 1, 3, 5, and 7 for SBNCOS for downstream quantification of Bd.

To evaluate the potential inhibitory effects of AE planktonic microorganisms on Bd growth, Bd was added to various media containing AE planktonic microorganisms, while media without AE microorganisms were used as the controls, as depicted in Fig. 1A. AE planktonic microorganisms were collected from the pond water from the 15 ponds in the SFEB region and one in SBNCOS as described in the General Methods below. Approximately 1 million Bd zoospores were exposed to 1 ml of pond water containing indigenous planktonic microorganisms in a 12-well plate at ambient laboratory temperature (20°C–23°C), without the use of a temperature-controlled incubator, for up to 1 week. For the SFEB samples, only the corresponding pond water was used as the test medium, and microorganism-depleted pond water served as the control. For the SBNCOS samples, three distinct media were tested to assess the influence of different microbial environments: (i) pond water containing indigenous planktonic microorganisms, (ii) 1% tryptone broth (TB), which is commonly used to support Bd growth and thus served as a growth-promoting reference medium, and (iii) sterile MQ water as a minimal-nutrient control. Both the supernatant and adherent material were collected for the Bd quantification on Days 1 and 7 for the SFEB samples, and Days 1, 3, 5, and 7 for the SBNCOS samples.

#### Experiment 3: Assessment of AE biofilms’ inhibitory impact on Bd

The techniques for evaluating the inhibitory effects of AE biofilms on Bd are depicted in Fig. 1B**.** AE biofilms attached to 12-well tissue culture plates were collected from nine ponds in the SFEB region and one in SBNCOS. These biofilms were gently rinsed with microorganism-depleted pond water using a low-pressure pipetting technique to remove loosely attached planktonic cells and debris. Care was taken to minimize shear stress by pipetting along the edges of the tissue culture well rather than directly onto the biofilm. Rinsed biofilms were then exposed to approximately 1 million Bd zoospores suspended in microorganism-depleted pond water obtained from the same site as the AE biofilms. For comparison, Bd was exposed to the SBNCOS biofilms in 1% TB or sterile MQ water. The adherent fraction was then subjected to quantitative analysis of Bd DNA within the biofilm on Days 1 and 7 for SFEB samples, and Days 1, 3, 5, and 7 for SBNCOS samples.

#### Experiment 4: Assessment of AE planktonic microorganisms’ effect on monolayer-associated Bd

The methods used to analyze the inhibitory effects of AE planktonic microorganisms on Bd monolayers are shown in the Fig. 1C. One million Bd zoospores were incubated in 1% TB medium in a 12-well tissue culture plate for 6 days to establish monolayers, which are predominantly composed of Bd zoosporangia rather than zoospores and resemble single-species biofilms [61]. Following incubation, the culture medium was aspirated, and the wells were gently rinsed with sterile MQ water to remove non-adherent Bd cells. Subsequently, 1 ml of pond water containing indigenous planktonic microorganisms, with or without 1% TB, was added to the wells containing the Bd monolayers and incubated at ambient temperature. Microorganism-depleted pond water, with or without 1% TB, served as the control medium. Bd monolayers were harvested for quantitative analysis on Days 1 and 7 from SFEB samples, and on Days 1, 3, 5, and 7 from SBNCOS samples.

### General methods

#### Filtration of pond water

The pond water underwent filtration procedures aimed at segregating large particles and extensively formed biofilm flocs, while preserving planktonic microorganisms such as bacteria, fungi, and similar entities. This was achieved by passing the water through a 40 *μ*m cell strainer, resulting in pond water containing indigenous planktonic microorganisms. Subsequently, to reduce the microbial load while preserving the natural chemical composition of the water, the pond water filtrate was further passed through a syringe filter with a pore size of 0.22 *μ*m to produce microorganism-depleted pond water. This filtration step substantially reduces bacterial and eukaryotic microorganisms, although it does not achieve complete sterilization (Fig. S3).

#### Culture of Bd

Bd (strain CJB4, isolated from *Rana sierrae* in 2011 from the California Sierra Nevada) was retrieved from cryogenic storage and maintained on 1% tryptone agar in 100 mm Petri dish at ambient temperature. Cultures were passaged weekly by transferring zoospores, collected from the agar surface to a fresh 1% tryptone agar plate at a 1:5 ratio. All experiments were conducted using cultures between passage numbers 25 and 40. Zoospores were harvested by adding sterile MQ water onto the agar plate, transferring into a falcon tube, and pelleting by centrifugation at 2000 rpm for 10 min. To eliminate residual tryptone agar, the pellet was washed once with the MQ water and recentrifuged. Zoospore counts were determined using a hemocytometer, and the zoospores were then ready for use in the experiments.

#### Quantification of Bd DNA

Following the Bd exposure to the AE microbiota in the various experiments as described above, the cells present in the supernatant were transferred into 1.5 ml Eppendorf tubes and pelleted via centrifugation, whereas those adhering to the plastic substrate were scraped using a cell scraper (Falcon™ Cell Scrapers, Cat. No. CLS353085, Corning, NY) and transferred to 1.5 ml Eppendorf tubes. Subsequent to sample collection, the pellet was resuspended in 100 *μ*L of PrepMan™ Ultra Sample Preparation Reagent (Life Technologies LTD, Warrington, UK), and Bd cell walls were disrupted using a Mini-BeadBeater-24 (BioSpec Products, Inc. Bartlesville, OK) at 2400 rpm for 1 min, then incubated in a 95°C heat block for 10 min and subsequently placed on ice for at least 2 min. The sample was then centrifuged at 10 000 rpm for 3 min, and the resulting DNA-containing supernatant was transferred to a new 1.5 ml Eppendorf tube. The DNA was further purified using GeneReleaser (BioVentures, Inc., Murfreesboro, TN) to remove potential inhibitor for the downstream quantitative PCR (qPCR) assay. Bd DNA was quantified using StepOne Plus real-time PCR following the protocol established by Boyle *et al.* [68]. For strain CJB4, which is representative of Sierra Nevada isolates, Dr Knapp and colleagues previously determined an average of 60 copies of the ITS1–5.8S rDNA region per zoospore [69]. Therefore, the zoospore equivalents (ZE) values can be converted to ITS copy numbers by multiplying them by 60. It is important to note that qPCR detects Bd DNA regardless of cell viability; thus, the presence of Bd DNA does not necessarily indicate the presence of live, infectious organisms.

### Statistical analyses

The reduction of Bd quantities in the experiments using samples from SFEB was calculated using the formula: ln(Bd at time *t*2​) − ln(Bd at time *t*1​), which we refer to as the Bd-inhibitory potency. Bd at time *t* refers to the number of ZE quantified by qPCR. All analyses were conducted using R version 4.4.1 (2024), with the stats package for ANOVAs, the nlme package for mixed-effects models, and the emmeans package for *post hoc* comparisons.

To examine the Bd-inhibitory potency of AE planktonic microorganisms in Experiment 2, distinct statistical approaches were employed based on sample sources. For the SFEB samples, a paired t-test was conducted with data paired by pond to compare the reduction of Bd under two experimental conditions: (i) Bd maintained in pond water containing indigenous planktonic microorganisms and (ii) Bd maintained in microorganism-depleted pond water (control). For the SBNCOS samples, a three-way ANOVA was performed on log-transformed Bd quantities. Predictor variables include day, presence or absence of a 1% TB supplement, water treatment (presence or absence of AE planktonic microorganisms in pond water and a sterile MQ water), and their interactions.

To evaluate the impact of AE biofilms on Bd in Experiment 3, for the SFEB samples, a paired t-test was used to compare log-transformed Bd quantities between Day 1 and Day 7. For the SBNCOS samples, a two-way ANOVA was performed with log-transformed Bd quantity as the response variable and day, medium, and their interaction as predictor variables.

The impact of SFEB planktonic microorganisms on Bd monolayers in Experiment 4 was assessed using a paired t-test to compare Bd*-*inhibitory potency under conditions with and without indigenous microorganisms. For SFEB planktonic microorganisms, analyses also included a planned pairwise comparison on Day 1 using a linear mixed-effects model, with log-transformed Bd quantity as the response variable, and day and presence or absence of AE planktonic microorganisms predictors. Ponds were included as a random effect to account for the paired nature of the data. For SBNCOS planktonic microorganisms, we used a three-way ANOVA on log-transformed Bd quantity, with predictor variables including day, presence or absence of AE planktonic microorganisms, presence or absence of a 1% TB supplement, and their interactions.

Finally, to compare the Bd-inhibitory efficacy between AE planktonic microorganisms and AE biofilms, a one-way ANOVA was conducted to analyze the reduction in Bd abundance (log-transformed Bd on Day 1 minus log-transformed Bd on Day 7) across four incubation conditions: pond water containing SBNCOS planktonic microorganisms, SFEB planktonic microorganisms, SBNCOS biofilms, or SFEB biofilms.

## Results

### In Experiment 1, AE biofilms but not planktonic microorganisms support the life cycle of Bd

**Figure 2:**
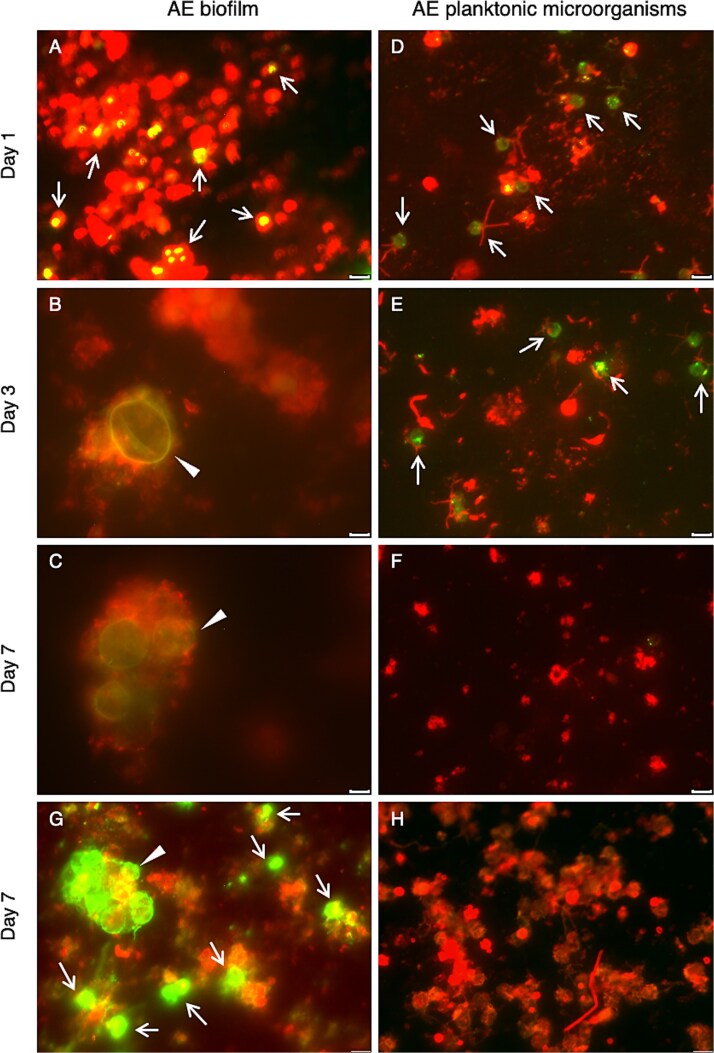
Fluorescent microscopic analysis of Bd life cycle. AE microbiota collected from SBNCOS pond was exposed to either green FL-Bd zoospores (**Panels A–F**) or unlabeled Bd zoospores (**Panels G and H**). For the FL-Bd samples, zoospores were directly visualized via green fluorescence. For the unlabeled Bd samples, zoospores were detected by immunostaining using a mouse monoclonal antibody against Bd, followed by an Alexa Fluor 488-conjugated goat anti-mouse IgG secondary antibody (green). Immunostaining was employed in these panels to distinguish newly released zoospores produced by biofilm-associated zoosporangia from the initially introduced zoospores. Glycoconjugates were detected by immunostaining using Alexa 594-conjugated Con A (red). Yellow indicates colocalization of Bd and Con A. Arrows mark Bd zoospores, and arrowheads denote Bd zoosporangia. Scale bar: 5 *μ*m for all images. The images shown are representative of consistent results obtained from three independent experiments using SBNCOS samples.

To investigate whether Bd can complete its life cycle in the presence of AE biofilms, FL-Bd zoospores were exposed to SBNCOS biofilms in the microorganism-depleted pond water. Immunofluorescent staining with Alexa-594 conjugated Con A revealed clustered, intense red fluorescence with a cloud-like pattern, indicative of abundant glycoconjugates within the extracellular matrix in the biofilms (Fig. 2**, panels A, C, E, and G;**  Fig. S4**, panels A, C, E, and G**). Remarkably, as early as 1 day post-incubation, the FL-Bd adhered to the biofilms, as evidenced by colocalization of FL-Bd and Con A in yellow (Fig. 2A). The green fluorescence on the FL-Bd cells remained detectable throughout the 7-day experimental period (Fig. 2**, panels C and E**). Notably, on Days 3 and 7, only FL-Bd zoosporangia (>5 *μ*m) were observed, while FL-Bd zoospores (<5 *μ*m) were undetectable, suggesting that the zoospores had developed into zoosporangia. The absence of fluorescent zoospores indicates that if release of zoospores from the FL-Bd zoosporangia occurred in this experiment, the new zoospores were not fluorescent (Fig. S5). To determine whether zoospores were being released from the zoosporangia during exposure to AE biofilms, we performed immunofluorescent staining using an anti-Bd monoclonal antibody, capable of detecting both zoospores and zoosporangia. On Day 7, both zoospores and zoosporangia exhibited high green fluorescence in the co-culture (Fig. 2G), indicating immunolabeling by the anti-Bd monoclonal antibody. This observation suggests that zoospores were produced from the zoosporangia and AE biofilms provide a supportive environment for the Bd life cycle.

In contrast, we examined the influence of SBNCOS pond water containing planktonic microorganisms on the Bd life cycle. Con A staining of the AE planktonic microorganisms revealed a dispersed, punctate red fluorescence pattern consistent with individual cell-surface carbohydrates (Fig. S4**, panels B, D, F, and H)**. The differences observed between panels F and H likely reflect natural microbial variation; however, neither exhibited the cloud-like distribution characteristic of biofilms. When FL-Bd zoospores were introduced into pond water containing planktonic microorganisms, the FL-Bd cells remained small (less than 5 *μ*m) on Days 1 and 3. By Day 7, the FL-Bd signal was negligible (Fig. 2B, D, F). Immunofluorescent staining did not detect Bd in the co-culture by Day 7 (Fig. 2H). This suggests that while Bd and AE planktonic microorganisms may exhibit a tendency to adhere to each other, the conditions did not support the development of zoosporangia.

### In Experiment 2, Bd DNA levels decreased significantly in the presence of aquatic environmental planktonic microorganisms

**Figure 3:**
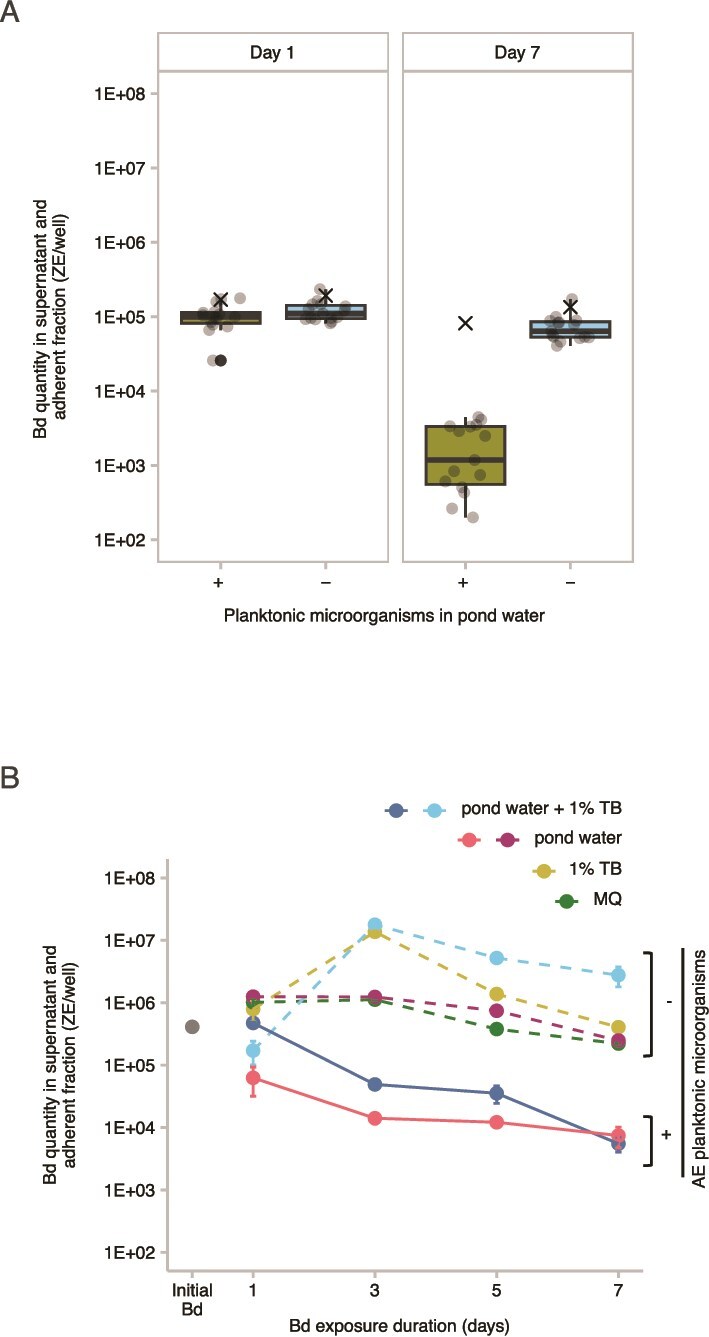
Comparison of Bd quantities in the presence and absence of AE planktonic microorganisms. (A) Boxplots show the Bd quantity in pond water from the SFEB ponds (*n* = 15), measured after 1 and 7 days of exposure, with the central line representing the median. The x-axis indicates the presence (+) or absence (−) of AE planktonic microorganisms. Gray-filled circles represent Bd quantities from individual ponds, black-filled circles denote outliers. × represents Bd in sterile MQ water without AE microorganisms. (B) A scatterplot illustrates the relationship between exposure duration (x-axis) and Bd quantity in SBNCOS pond water (y-axis). Solid lines indicate Bd zoospores exposed to AE planktonic microorganisms, dashed lines represent controls without microorganisms. Data points indicate mean Bd quantity from triplicate measurements; error bars represent the standard error of the mean.

The pond water containing indigenous planktonic microorganisms showed a significantly greater reduction in Bd between Day 1 and Day 7 compared to that in the microorganism-depleted pond water (*n* = 15, paired t-test, t = 12.4, df = 14, *P* < .0001; Fig. 3A). Remarkably, AE planktonic microorganisms from each of the 15 ponds in the SFEB region exhibited Bd-inhibitory effect (Fig. S6). Figure S6 shows results from individual ponds, complementing the pooled data in Fig. 3A and confirming the reproducibility of this pattern. These findings strongly suggest that AE planktonic microorganisms present in the pond water exert an inhibitory function on Bd proliferation. Furthermore, we also observed a statistically significant difference in Bd quantity after 7 days in the presence of SBNCOS planktonic microorganisms compared to that in the absence of the AE microorganisms, regardless of the culture media used (Fig. 3B). A three-way ANOVA revealed that there was a statistically significant difference in Bd quantity across days (F_3, 48_ = 45.61, *P* < .0001), presence of 1% TB (F_1,48_ = 81.13, *P* < .0001), water treatment, which compares pond water with and without AE microorganisms and a MQ water (F_2,48_ = 442.25, *P* < .0001). There were also significant interactions between the effects of day and 1% TB (F_3, 48_ = 15.08, *P* < .0001), between day and water treatment (F_6, 48_ = 24.05, *P* < .0001), and the third-order interaction between day, presence of 1% TB, and water treatment (F_6, 48_ = 15.82, *P* < .0001). A Tukey–Kramer *post hoc* analysis with a 95% family-wise confidence level revealed several key findings. The amount of Bd peaked on the third day before declining thereafter (Fig. S7A). The presence of 1% TB was associated with a higher abundance of Bd (Fig. S7B), while pond water containing planktonic microorganisms exhibited the lowest Bd levels (Fig. S7C). Notably, significant interactions were observed between time and the presence of 1% TB (Fig. S7D) as well as between time and the presence of AE planktonic microorganisms (Fig. S7E), underscoring the combined influence of these variables on Bd dynamics over time. Third-order comparisons are presented in Fig. S7F. Cumulatively, our data indicate that the Bd-inhibitory function of AE planktonic microorganisms is robust, regardless of the media used for the Bd culture.

### In Experiment 3, Bd DNA was reduced in the presence of aquatic environmental biofilms

**Figure 4:**
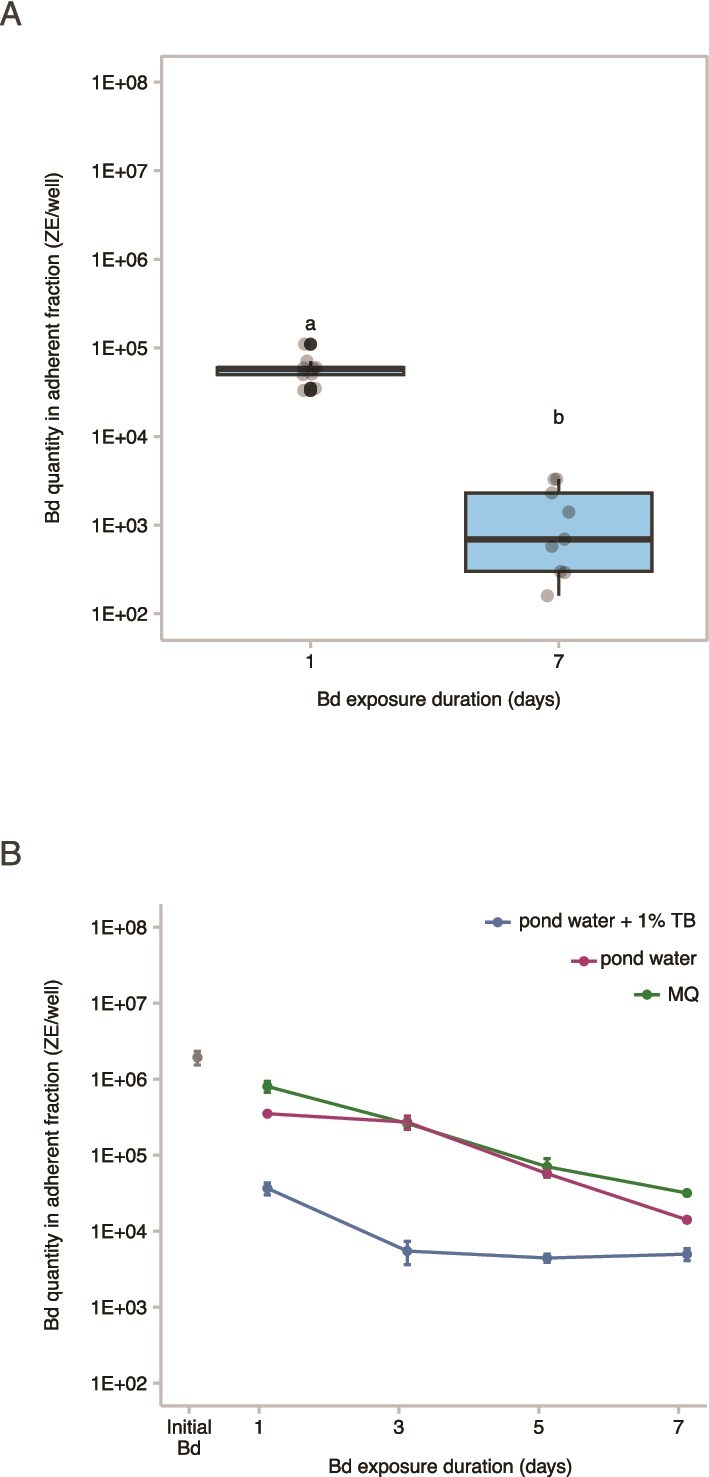
Bd quantity in the presence of AE biofilms. (A) Boxplots illustrate the Bd quantity in SFEB biofilms (*n* = 9), measured after 1 and 7 days of exposure. The central line represents the median. Gray-filled circles represent Bd quantities from individual biofilms; black-filled circles denote outliers. The x-axis denotes the exposure duration. Letters (a, b) indicate statistically significant differences based on pairwise comparisons (*P* < .0001). (B) A scatterplot displays the relationship between exposure duration (x-axis) and Bd quantity in SBNCOS biofilms (y-axis). Data points represent the mean Bd quantity within AE biofilms, with error bars indicating the mean ± standard error from triplicate measurements.

Twenty-four hours post-exposure of Bd zoospores to AE biofilms in 12-well tissue culture plates, an increase in Bd quantity was observed within the AE biofilms collected from SFEB ponds (Fig. S8). This suggests that Bd zoospores initially adhere to the AE biofilms during the first 24-h period. Subsequently, a significant decrease in Bd quantity within the AE biofilms was detected on Day 7 compared to Day 1 (paired t-test, df = 8, t = 10.10, *P* < .0001; Fig. 4A). Figure S8 presents results from individual ponds, complementing the summarized outcomes in Fig. 4A, and demonstrating the similarity of this pattern. As shown in Fig. 4B, a two-way ANOVA revealed that, in the SBNCOS pond water experiment, there was a statistically significant difference in Bd quantity across days (F_3, 24_ = 110.1, *P* < .0001), media (F_2,24_ = 246.6, *P* < .0001), and the interaction between the effects of day and media were also significant (F_6, 24_ = 10.1, *P* < .0001). *Post hoc* analyses revealed significant pairwise differences, as determined by a Tukey–Kramer test with 95% confidence intervals (Fig. S9). Across time, we found that the largest amount of Bd was observed on Day 1, and each following day had significantly less Bd (Fig. S9A) When comparing across media, the greatest Bd inhibition was observed with the addition of 1% TB, followed by pond water, and MQ water exhibiting the weakest inhibitory effect (Fig. S9B). This suggests that the AE biofilm’s inhibitory capacity was enhanced by the 1% TB supplement. There were statistically significant interactions between incubation time and media type, underscoring the importance of both media composition and duration of exposure in influencing Bd reduction within the AE biofilms (Fig. S9C).

### Bd-inhibitory potency of the AE biofilms is comparable to that of the AE planktonic microorganisms

**Figure 5:**
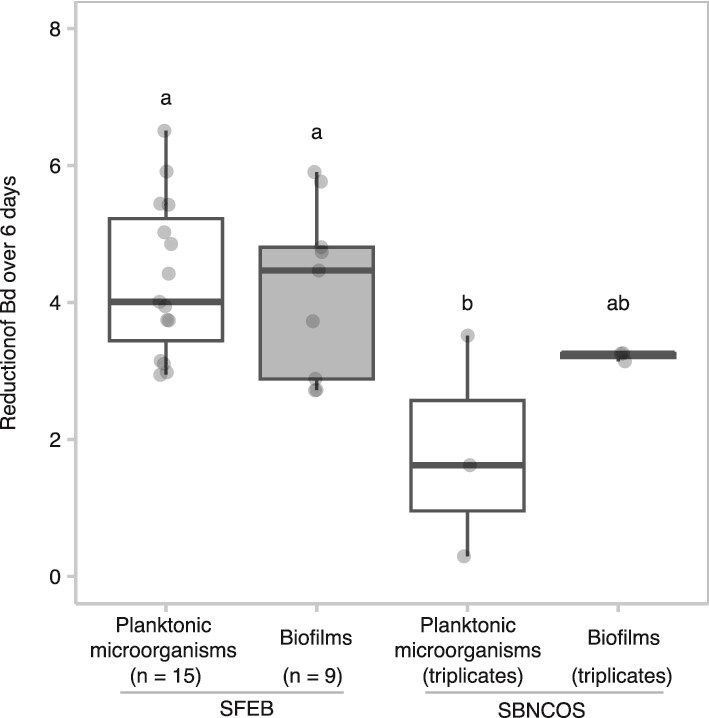
Comparison of the reduction rate of Bd treated with AE planktonic microorganisms versus AE biofilms. Boxplots indicate the reduction rate of Bd over the 6 days with a central line representing the median. Gray-filled circles represent individual data points. Different lowercase letters above the boxes indicate results of Tukey’s *post hoc* pairwise comparisons (95% confidence interval, α = .05). Groups sharing at least one letter are not significantly different; groups with no letters in common are significantly different. For example, bars labeled “a” and “ab” are not significantly different, while “a” and “b” indicate a statistically significant difference.

We compared the Bd-inhibitory potency of AE biofilms to that of AE planktonic microorganisms. Overall, Bd reduction significantly differed between the experiments (one-way ANOVA F_3, 26_ = 4.42, *P* = .012). Figure 5 illustrates that within the SFEB ponds, Bd reduction by AE planktonic microorganisms was statistically similar to that by AE biofilms (Tukey *post hoc* test, *P* = .99). A similar trend was observed in the SBNCOS pond: although AE biofilms exhibited a slightly greater mean Bd reduction than AE planktonic microorganisms, the difference was not statistically significant (Tukey *post hoc* test, *P* = .47). Between-location comparisons revealed that AE biofilms from SFEB tended to be more inhibitory than those from SBNCOS, although the difference was not statistically significant. In contrast, AE planktonic microorganisms from SFEB demonstrated significantly greater Bd-inhibitory potency than those from SBNCOS. Collectively, these findings indicate that AE biofilms and planktonic microorganisms exhibit comparable Bd-inhibitory potency within each location. However, site-specific differences, particularly in the efficacy of AE planktonic microorganisms, suggest underlying ecological variation that merits further investigation.

### In Experiment 4, Bd monolayers exhibit a rapid reduction of Bd in the presence of AE planktonic microorganisms

**Figure 6:**
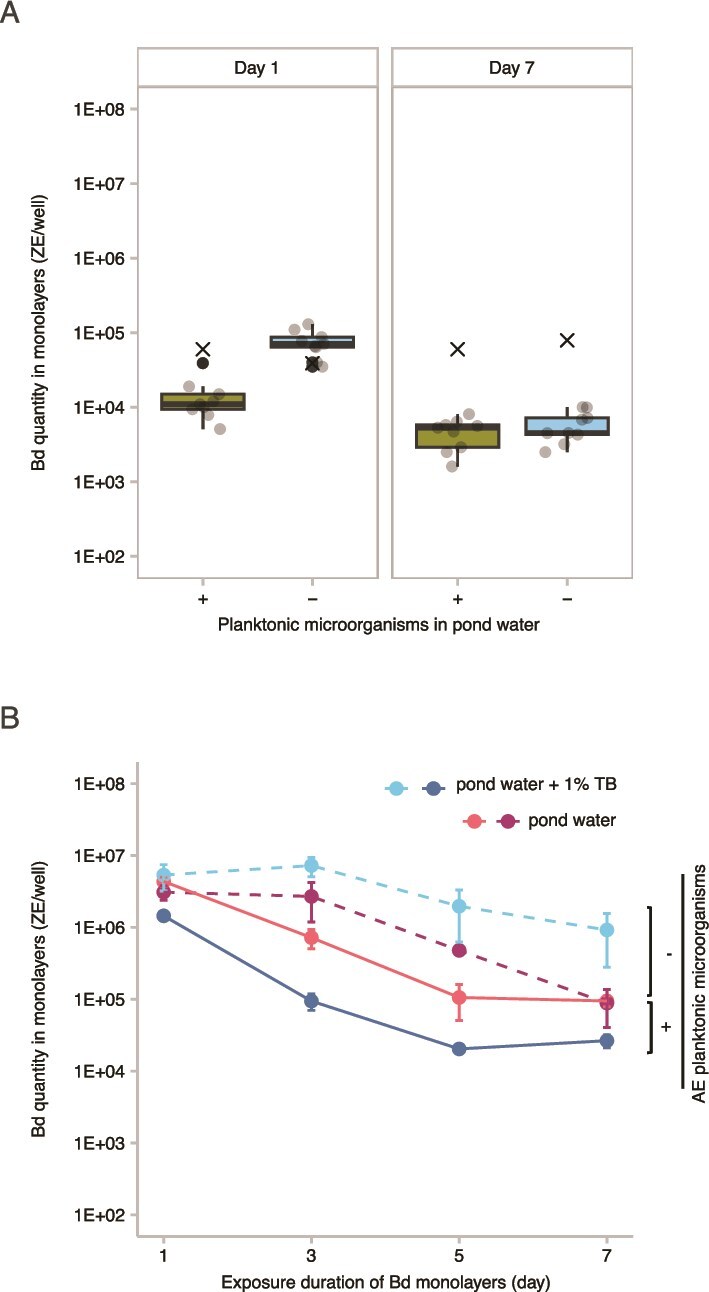
Comparison of Bd quantities in monolayers in the presence or absence of AE planktonic microorganisms. (A) Boxplots depict the Bd quantity in monolayers exposed to pond water from SFEB ponds (*n* = 9), measured after 1 and 7 days of exposure. The central line represents the median. Gray-filled circles represent Bd quantities from individual ponds; black-filled circles denote outliers. “×” represents Bd in sterile MQ water without AE microorganisms. The x-axis shows the presence (+) or absence (−) of AE planktonic microorganisms. (B) A scatterplot illustrates the relationship between exposure duration (x-axis) and Bd quantity within monolayers in SBNCOS pond water (y-axis). Solid lines indicate Bd monolayers exposed to AE planktonic microorganisms, while dashed lines represent controls without AE microorganisms. Data points show the mean Bd quantity from triplicate measurements, with error bars representing the standard error of the mean.

Following exposure of Bd monolayers to the pond water collected from the SFEB ponds, Bd abundance was significantly reduced after 1 day in the presence of AE planktonic microorganisms compared to microorganism-depleted pond water (linear mixed-effects model and planned pairwise comparison, *P* < .0001) (Fig. 6A). Figure 6A displays the overall response across nine SFEB ponds, with Fig. S10 detailing each pond’s data, all showing a clear inhibitory effect of AE planktonic microorganisms on Bd monolayers. This finding indicates that AE planktonic microorganisms exert an inhibitory effect on Bd monolayers. Moreover, Bd monolayers maintained in microorganism-depleted pond water exhibited a significantly greater reduction in Bd abundance between Day 1 and Day 7 compared to those in microorganisms-containing pond water (paired t-test, t = −5.4, df = 8, *P* < .001), however this appears to stem from the initial suppression of Bd on Day 1 in the presence of AE planktonic microorganisms. For the SBNCOS samples, a three-way ANOVA revealed that there was a statistically significant difference in Bd quantity across days (F_3, 32_ = 45.34, *P* < .0001), the presence of AE planktonic microorganisms (F_1,32_ = 54.84, *P* < .0001), but no significant effect of the presence of 1% TB supplement by itself (*P* > .05) (Fig. 6B and Fig. S11). There were significant interactions between the effects of day and the presence of planktonic microorganisms (F_3, 32_ = 5.90, *P* = .003), and the interaction between the effects of the presence of planktonic microorganisms and the presence of 1% TB supplement (F_1, 32_ = 26.28, *P* < .0001) (Fig. 6B and Fig. S11). The addition of 1% TB enhanced the inhibitory effect of AE planktonic microorganisms, as evidenced by the lower Bd quantities observed in the pond water with planktonic microorganisms supplemented with 1% TB, compared to pond water with planktonic microorganisms alone (Fig. 6B and Fig. S11D). Conversely, in the absence of AE microorganisms, the addition of 1% TB resulted in higher Bd quantities compared to that without 1% TB (Fig. S11D). These findings further confirm the inhibitory effect of AE planktonic microorganisms on Bd monolayers, in agreement with the results presented in Fig. 6A. No significant interactions were found between day and the presence of 1% TB, and there was no significant third-order interaction among day, the presence of AE planktonic microorganisms, and presence of 1% TB, did not have a significant effect (Tukey *post hoc* test, *P* > .05).

## Discussion

Our study provides new insights into the context-dependent role of AE biofilms in modulating Bd dynamics in the aquatic environment. We demonstrated that AE biofilms, but not planktonic microorganisms, support the completion of Bd’s life cycle in the short term, while AE microbes in both biofilms and planktonic forms exhibit comparable inhibitory effects on Bd growth. These findings highlight the dual function of AE biofilms as both supporters and suppressors of Bd, and underscore their potential influence on environmental transmission dynamics and host-pathogen-microbiota interactions.

To our knowledge, this is the first evidence that AE biofilms provide a potential niche allowing Bd to complete its life cycle, at least transiently, in the absence of amphibian hosts*.* This contrasts with planktonic microorganisms, which do not allow Bd’s life cycle progression beyond the zoospore stage. The biofilms matrix, rich in extracellular polymeric substances, likely offers a permissive microenvironment that promotes Bd adhesion, nutrient acquisition and retention, and protection from environmental stressors, thereby allowing zoosporangia formation [59, 61, 70]. These biofilm-associated habitats could increase opportunities for environmental exposure, particularly for tadpoles grazing on benthic substrates where Bd may be embedded in biofilms, posing a higher risk for amphibians to become infected by the pathogen.

However, the ability of AE biofilms to support Bd development appears to be limited in duration. We observed a decline in Bd quantity within 1 week, suggesting that this support is transient. This aligns with previous findings reporting no Bd survival beyond 3 weeks in biofilms [54]. This limited persistence of Bd in AE biofilms may still facilitate indirect environmental transmission but does not imply that Bd can maintain ecological independence from amphibian hosts.

During our AE biofilm sampling, amphibians and Bd-positive individuals were observed at both the SFEB and SBNCOS sites. We speculate that amphibians may influence biofilm communities through direct interactions such as tadpole grazing, secretion of antimicrobial peptides [44, 71], or deposition of skin-associated microbiota [72, 73]. In addition, tadpole feces, rich in microorganisms and nutrients, likely represent an important pathway by which tadpoles locally affect biofilm composition. These interactions could reinforce or disrupt the Bd-inhibitory capacity of biofilms, creating a dynamic feedback loop between host behavior and pathogen regulation. Amphibian-biofilm interactions thus represent an important yet understudied component of chytridiomycosis ecology. *In situ* studies that incorporate amphibian hosts and natural environmental complexity, along with long-term monitoring of host–pathogen–microbiota dynamics, are needed to clarify their role in Bd transmission in the wild.

The functional role of biofilms may shift over time, from initially permissive to ultimately inhibitory, as microbial composition evolves or microbial activity intensifies. Previous studies have linked Bd inhibition to factors such as biofilm thickness, the abundance of Bd-inhibitory bacteria, and overall microbial diversity [53]. Our study did not include microbial community profiling, which limits our ability to determine whether Bd suppression was driven by specific microbial taxa, community structure, or properties of the biofilm matrix. Future studies incorporating microbial taxonomic profiling are needed to address this gap.

To further assess AE microbial inhibition, we used Bd monolayers as an *in vitro* model [61]. Although not a natural developmental stage of Bd, monolayers serve as a proxy for dense and adherent growth forms. In this study, the low Bd quantity observed on Day 1 in the presence of SFEB pond planktonic microorganisms may reflect a rapid inhibitory effect, suggesting that monolayer Bd is more susceptible than free zoospores. Bd monolayers, enriched in zoosporangia, may confer resistance to alkaline conditions and certain plant-derived antifungal compounds, such as Allicin [61], whereas another study found zoosporangia more susceptible than zoospores to the heavy metals [74]. These findings indicate that Bd’s vulnerability varies with growth form and environmental context.

Our nutrient supplementation experiments revealed that 1% TB supplementation enhanced the Bd-inhibitory effect of AE microbiota. We interpret this as evidence of competitive suppression, where AE biofilms and planktonic microorganisms outcompete Bd for shared resources under conditions of high resources availability. Similar nutrient-driven community shifts have been observed in algal–Bd–tadpole co-culture systems, where nitrogen and phosphorus enrichment promoted algal growth, indirectly suppressing Bd and mitigating its negative effects on tadpoles [75]. Although direct competition between algae and Bd was not assessed in that study, the findings suggest that nutrient enrichment can alter microbial community structure in ways that reduce Bd proliferation.

Finally, our comparison of Bd-inhibitory potency between AE biofilms and planktonic microorganisms showed similar levels of suppression within each location, suggesting they can both suppress Bd through similar mechanisms, such as producing antifungal metabolites [76, 77]. However, we observed that AE planktonic microorganisms from SFEB exhibited stronger Bd inhibition than those from SBNCOS, while AE biofilms from SFEB also trended more inhibitory than those from SBNCOS. These site-specific and geographic differences may reflect variation in microbial composition, micro-environmental conditions, or nutrient availability. Additionally, although our study distinguishes between surface-attached biofilms and free-living planktonic microorganisms, we acknowledge that biofilms naturally release planktonic cells into their surrounding environment. Thus, inhibitory effects observed in our biofilm experiments could result, in part, from planktonic cells shed from biofilms. Future studies should aim to experimentally distinguish the specific contributions of each microbial form. Taken together, our findings raise the potential for microbiota-based strategies to mitigate chytridiomycosis by leveraging both biofilm-associated and free-living microbes.

This study has a few methodological limitations worth noting. First, we used 0.22 *μ*m filtration to generate microorganism-depleted pond water while preserving natural water chemistry. Although this approach removes most bacteria, protozoa, and algae, it does not exclude ultra-small and filterable microorganisms [78]. Some of these residual entities may influence Bd dynamics. For instance, recent work has shown that certain mycoviruses in the *Circoviridae* family can infect Bd and modulate its growth and virulence [79]. However, consistent patterns across replicates suggest any such effects were likely limited. Future work could incorporate methods to better assess residual microbial presence. Second, our qPCR-based quantification may reflect differences in DNA extraction efficiency between life stages of Bd. Although we used PrepMan™ Ultra with bead-beating to improve lysis of zoosporangia, some underestimation of the zoosporangia stage cannot be ruled out. Therefore, reductions in Bd quantity, particularly under biofilm conditions, may reflect primarily decreased zoospore abundance. Improved extraction protocols capable of equally capturing both life stages would strengthen future analyses. Lastly, our zoospore harvesting method did not include filtration to remove residual zoosporangia. Microscopy observations confirmed only minimal presence of zoosporangia in the zoospore inocula. While this suggests that the majority of cells were indeed zoospores, we acknowledge that the presence of any remaining zoosporangia may have introduced minor bias in interpreting zoospore-specific trends. However, we consider this potential bias to be minimal and unlikely to substantially alter the overall conclusions of our study.

## Conclusion

This study suggests that AE biofilms play a complex, context-dependent role in Bd dynamics. While AE biofilms uniquely support Bd’s life cycle in the absence of amphibian hosts, both biofilms and planktonic microorganisms exhibit comparable Bd-inhibitory effects. These contrasting outcomes suggest that AE microbiota may influence Bd dynamics through multiple, potentially competing processes. Although the specific pathways remain to be investigated and our findings are based on controlled laboratory conditions, these findings highlight the ecological relevance of biofilm-associated habitats and suggest that microbiota-based approaches could inform future strategies for mitigating chytridiomycosis in amphibian populations.

## Supplementary Material

Supplementary_Information_for_Chen+_et_al_ycaf185

## Data Availability

All data and analysis scripts associated with this work will be publicly and freely available through https://github.com/cnordheim-maestas/chen-et-al-2025.
