# Supplementary material for: Double-edged effects of aquatic environmental biofilms on Batrachochytrium dendrobatidis growth and inhibition
Source: ISME Commun. 2025 Oct 16;5(1):ycaf185. doi: 10.1093/ismeco/ycaf185 (PMC12599316; doi:10.1093/ismeco/ycaf185)
Supplement: Supplementary_Information_for_Chen+_et_al_ycaf185 [file supplementary_information_for_chen+_et_al_ycaf185.docx]

**Supplementary Information for**

**Chen *et al*. (2025) “Double-Edged Effects of Aquatic Environmental Biofilms on *Batrachochytrium dendrobatidis* Growth and Inhibition” *ISME Communications***

Renwei Chen^1*^, Caitlin L. Nordheim-Maestas^2^, Cheryl J. Briggs^1, 2^

^1^Marine Science Institute, ^2^Ecology, Evolution, and Marine Biology, University of California Santa Barbara, California, USA

*Correspondence: Renwei Chen

Postal address: Marine Science Institute, Building 520, Room 4001, Floor 4L, University of California Santa Barbara, Santa Barbara, CA 93106

E-mail address: renwei@ucsb.edu

This file includes supplementary figures S1 to S11 and table S1.

Figure S1


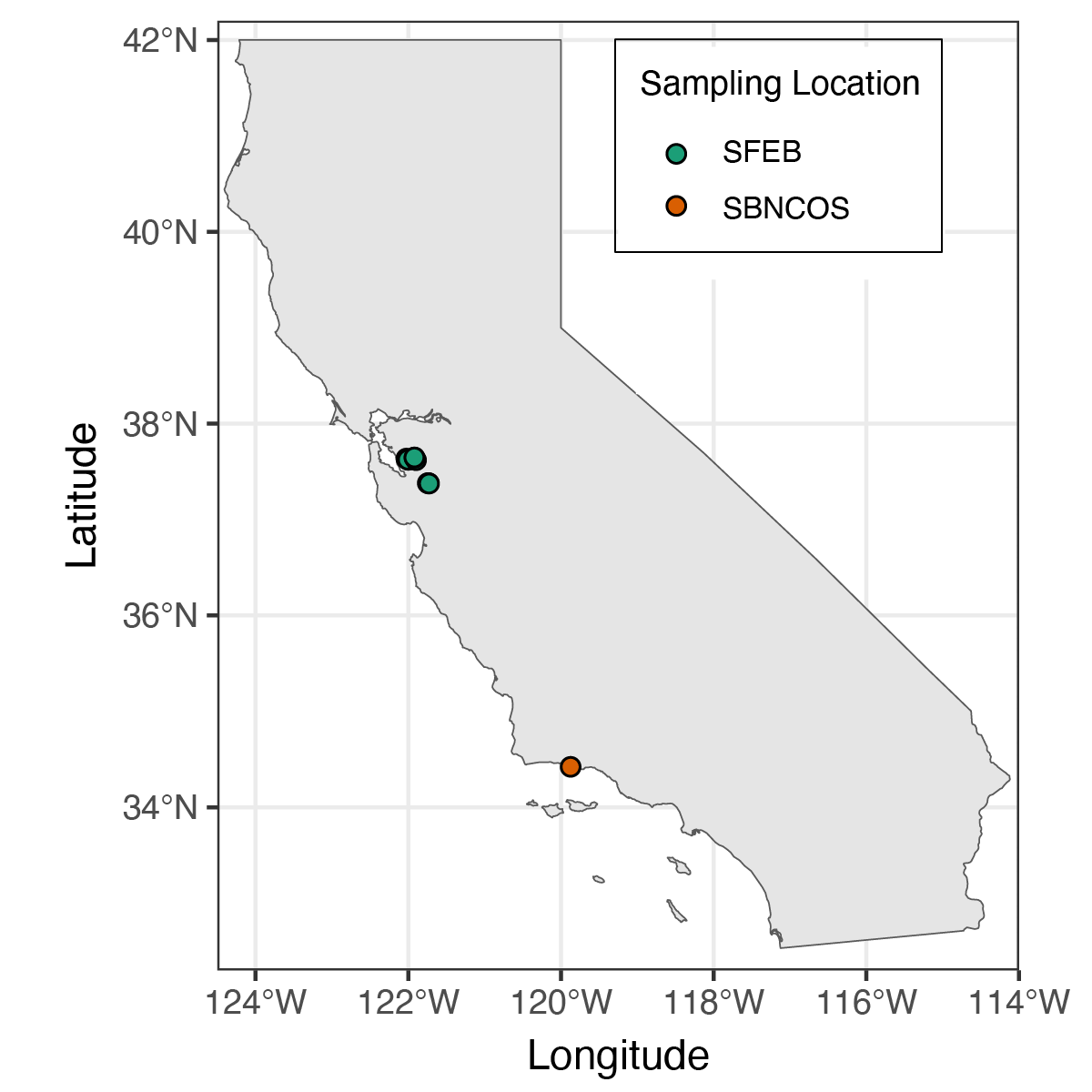


**Figure S1. Georeferenced map of pond sampling locations in California.** Map showing the geographic distribution of ponds sampled for aquatic environmental microbiota in two regions: and the San Francisco East Bay region (SFEB) and Santa Barbara North Campus Open Space (SBNCOS). Sampling locations are marked with latitude and longitude coordinates to illustrate the spatial range of the study.

**Table S1**. Amphibian Bd Sampling Summary, Detection, and Environmental Conditions

| Site | Species | Date | Swabbed (n) | Bd+ (n) | Prevalence (%) | Mean Bd Load (ZE) | Individuals Observed | Water Temp (°C) |
| --- | --- | --- | --- | --- | --- | --- | --- | --- |
| CABIN | *A. boreas* | 05/06/2022 | 1 | 0 | 0 | NA | 1 | 20 |
| CABIN | *P. regilla* | 05/06/2022 | 1 | 1 | 100 | 1,320 | 2 | 20 |
| CABIN | *R. catesbeiana* | 05/06/2022 | 4 | 4 | 100 | 299 | 4 | 20 |
| GDPND005 | *P. regilla* | 05/07/2022 | 1 | 1 | 100 | 1 | 1 | 21 |
| GDPND006 | *P. regilla* | 05/07/2022 | 2 | 0 | 0 | NA | 3 | 24 |
| GDPND009 | *P. regilla* | 05/07/2022 | 2 | 0 | 0 | NA | 2 | 25 |
| GRAMPS | *A. boreas* | 05/06/2022 | 1 | 0 | 0 | NA | 1 | 21 |
| GRAMPS | *P. regilla* | 05/06/2022 | 4 | 0 | 0 | NA | 4 | 21 |
| SBNCOS | *P. regilla* | 02/03/2021 | 6 | 1 | 17 | 731 | NA | NA |
| PRPND009 | *P. regilla* | 05/08/2022 | 5 | 0 | 0 | NA | 5 | 20 |
| PRPND010 | *P. regilla* | 05/08/2022 | 9 | 4 | 44 | 10,038 | 13 | 18 |
| WEST | *P. regilla* | 05/08/2022 | 10 | 0 | 0 | NA | 30 | 24 |

Note: NA = not available; ZE = zoospore equivalents.

Figure S2


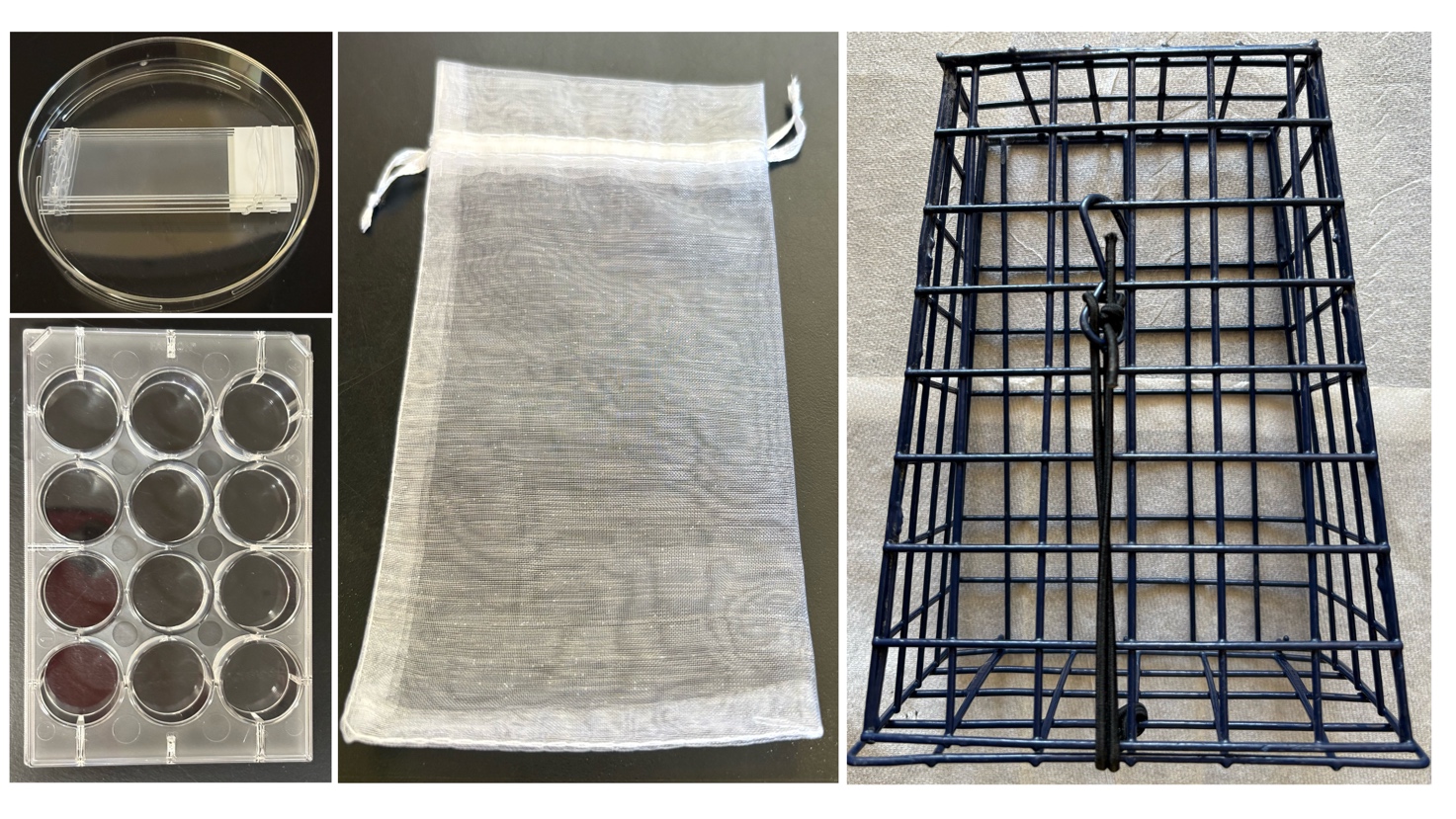


**Figure S2. Device for in situ collection of aquatic environmental biofilms from ponds.** The setup consisted of a 12-well tissue culture plate (Fisherbrand™, Cat. No. FB012928, Thermo Fisher Scientific, MA) and a stack of microscope slides with approximately 1 mm spacing between each slide (Superfrost™ Plus, Fisherbrand™, Cat. No. 12-550-15, Fisher Scientific, PA) placed within a Petri dish to promote microbial attachment. These components were enclosed in a nylon mesh pouch and housed within a protective cage to anchor the setup in the pond. The device was submerged in the pond for one week to allow natural biofilm development and then carefully retrieved and transported to the laboratory while fully immersed in water from the same sampling site to preserve microbial integrity.

Figure S3


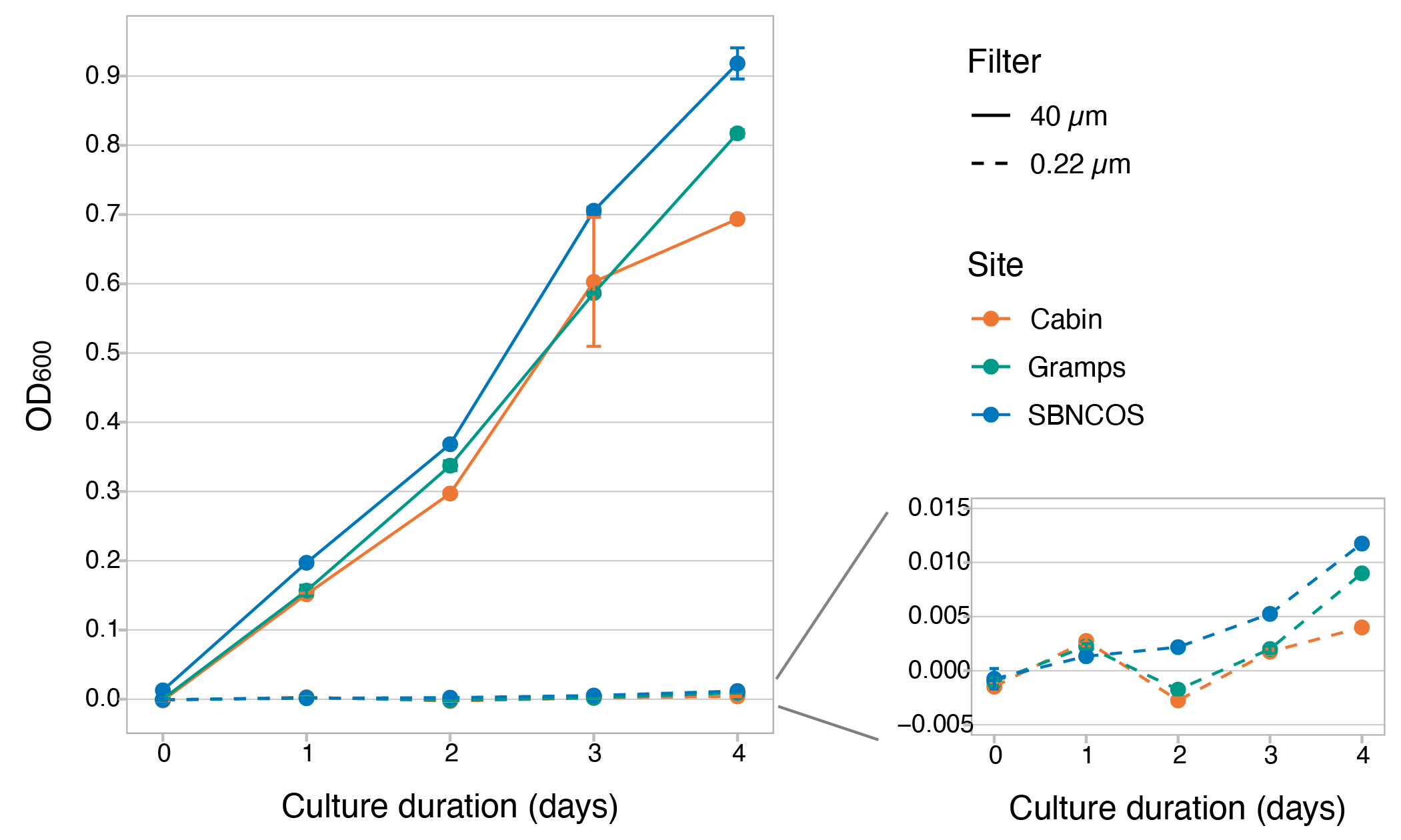


**Figure S3. Microbial growth in 1% tryptone broth following filtration of pond water samples.** Pond water was collected from three locations: Cabin and Gramps in San Francisco East Bay region, and SBNCOS (Santa Barbara North Campus Open Space). Each sample was first filtered through a 40 µm cell strainer. Half of each 40 µm filtrate was subsequently filtered through a 0.22 µm syringe filter. All filtrates were mixed 1:1 with 2% tryptone broth (TB), resulting in a final concentration of 1% TB in 50 mL Falcon tubes, and incubated at ambient temperature (20–23°C). Optical density at 600 nm (OD₆₀₀; y-axis) was measured every 24 hours over 4 days (x-axis) to assess microbial growth. Solid lines represent samples filtered only through the 40 µm cell strainer; dashed lines represent samples further filtered through the 0.22 µm syringe filter. All 40 µm-filtered samples showed progressive microbial growth, reaching peak OD₆₀₀ values by Day 4. In contrast, 0.22 µm-filtered samples exhibited negligible growth throughout the incubation period, indicating that 0.22 µm filtration effectively removed most viable microorganisms from the pond water. Data points represent the mean OD₆₀₀ values, and error bars indicate the standard error of the mean from four replicate measurements.

Figure S4


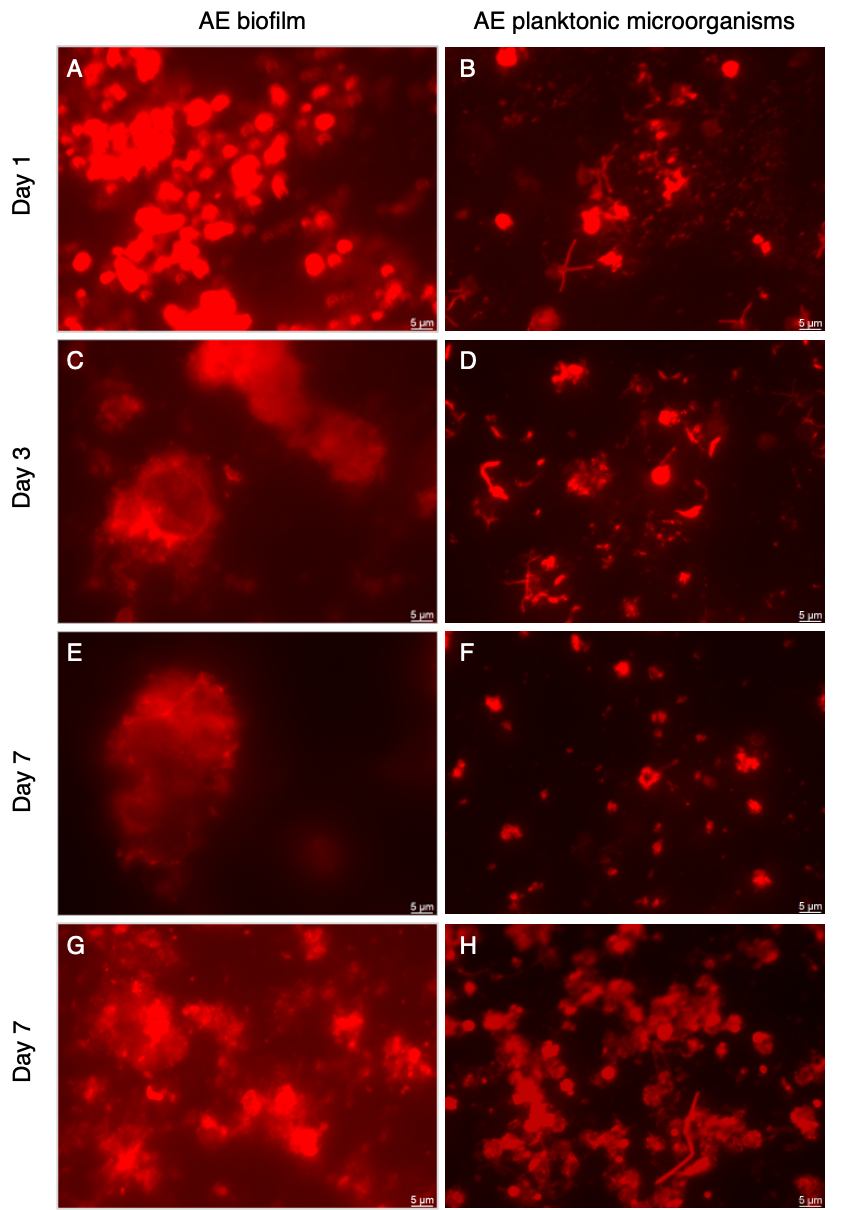


**Figure S4. Concanavalin A staining of aquatic environmental (AE) microbial communities exposed to Bd zoospores**. Glycoconjugates were visualized using Alexa 594–conjugated Concanavalin A (Con A; red). In AE biofilms exposed to Bd zoospores (A, C, E, G), Con A staining appeared clustered and cloud-like, indicating abundant extracellular polysaccharide matrices. In contrast, AE planktonic microorganisms exposed to Bd zoospores (B, D, F, H) exhibited dispersed, punctate staining patterns consistent with individual cell-surface carbohydrates. The differences between panels F and H likely reflect natural microbial variation, but neither displayed the cloud-like pattern characteristic of biofilms. Scale bars: 5 µm for all images.

Figure S5

**
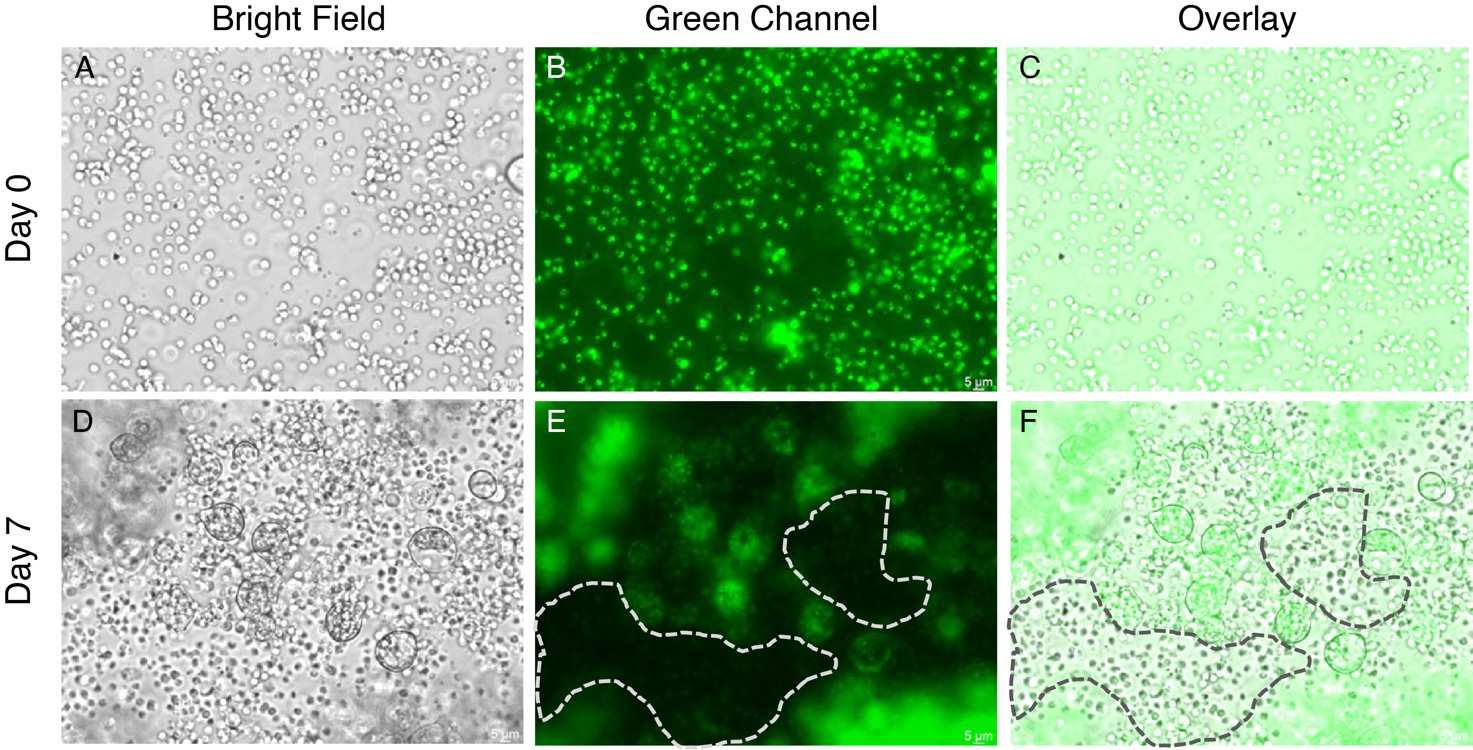
**

**Figure S5. Newly released zoospores are non-fluorescent.** Green fluorescent-labeled (FL)-Bd zoospores were imaged on day 0 (Panels A–C) and after seven days of culture in 1% tryptone broth medium at ambient temperature (Panels D–F). On day 0, nearly all zoospores exhibited bright green fluorescence (Panels B and C). On day 7, bright-field microscopy revealed both zoospores (≤ 5 µm) and zoosporangia (> 5 µm) (Panel D); however, the fluorescence was retained exclusively in zoosporangia (Panel E); while zoospores, outlined by dashed lines, were non-fluorescent (Panels E and F). This confirms that, after seven days of culture, these zoospores were produced from the zoosporangia rather than the original FL-labeled population. Scale bar: 5 µm for all images.

Figure S6

**
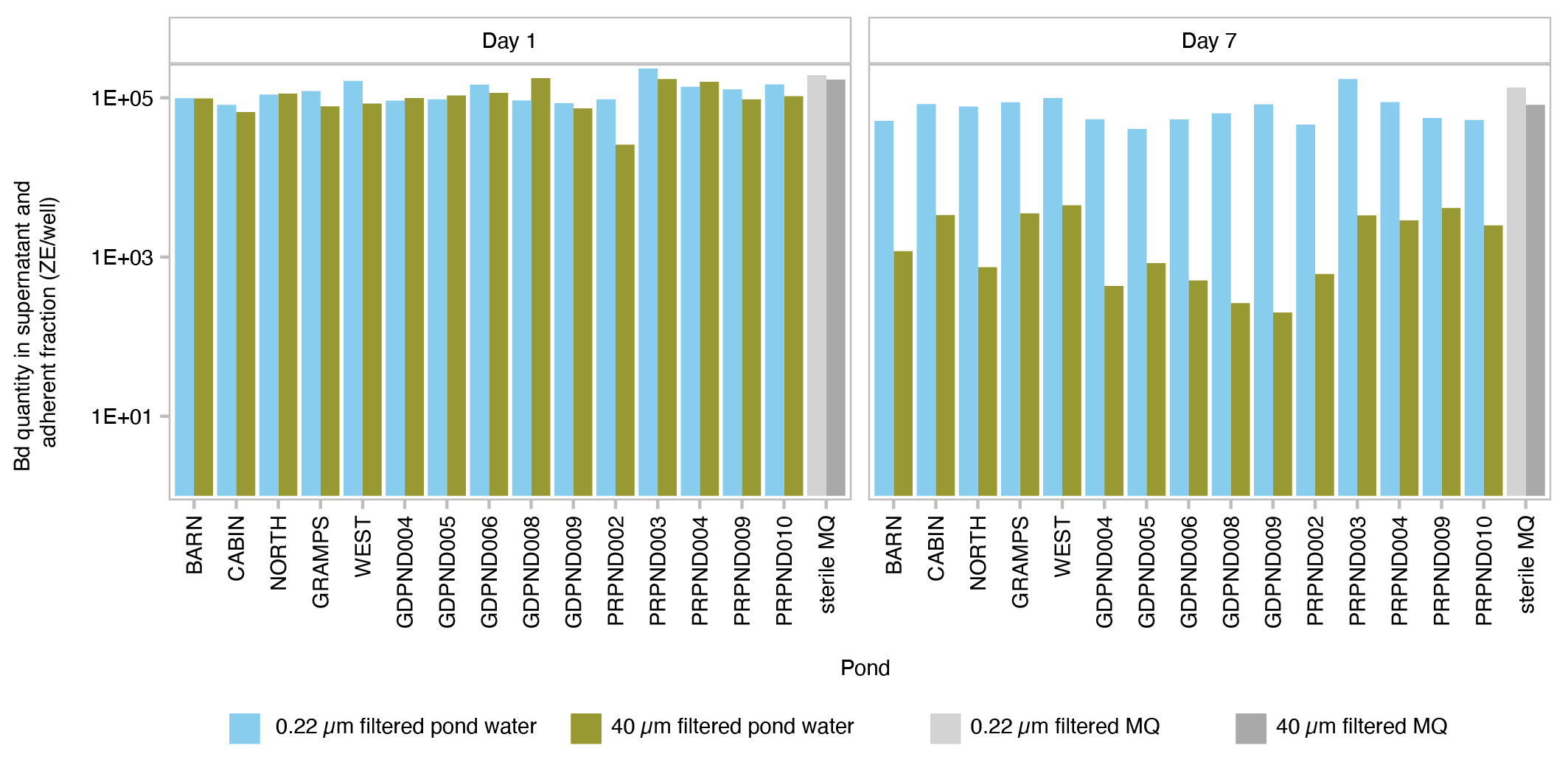
**

**Figure S6. Quantification of Bd in the presence or absence of AE planktonic microorganisms.** Bd quantities (zoospore equivalents [ZE] per well) in both supernatant and adherent fractions of the sample were measured on days 1 and 7 following exposure to pond water collected from 15 sites in the San Francisco East Bay region. Each bar represents the Bd quantity per well for a given pond sample at a given timepoint. Values were not pooled across treatments. The x-axis shows individual ponds and the sterile Milli-Q water (MQ), while the y-axis displays Bd quantity on a logarithmic scale. Filtration through a 40 µm filter retained AE planktonic microorganisms, whereas the 0.22 µm filter excluded them. The data highlight temporal and microbial condition-dependent variation in Bd quantities.

Figure S7


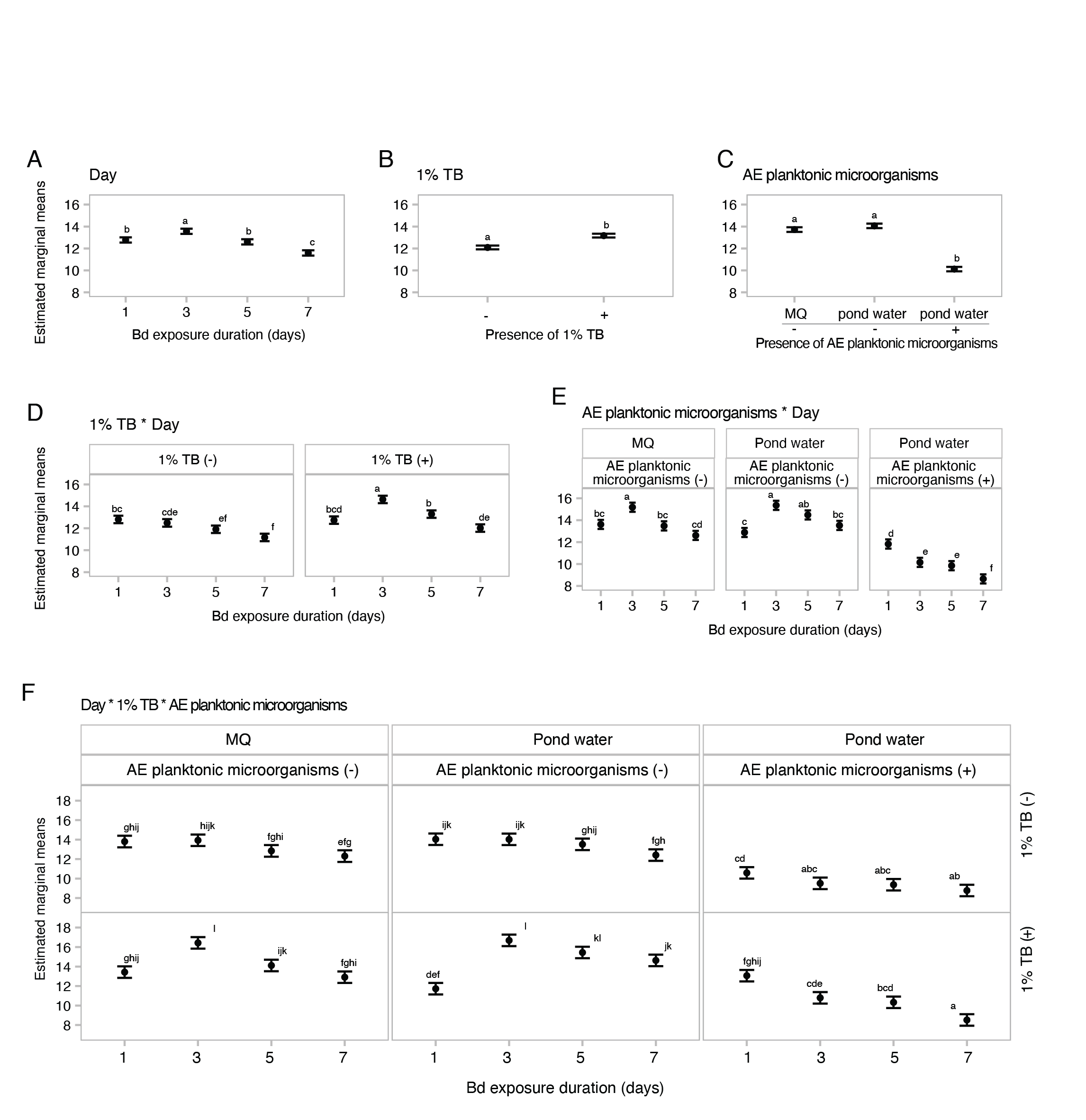


**Figure S7. Pairwise comparisons of Bd quantity across exposure duration, nutrient availability, and presence of aquatic environmental (AE) planktonic microorganisms.** Data are shown on a logarithmic y-axis. (A) Estimated marginal means of Bd quantity measured on days 1, 3, 5, and 7. (B) Effect of 1% tryptone broth (TB) on Bd quantity, comparing treatments with (+) and without (-) TB. (C) Impact of AE planktonic microorganisms on Bd quantity; MQ denotes the sterile Milli-Q water. (D) Interaction between 1% TB and sampling day, showing how nutrient availability affects temporal changes in Bd growth. (E) Interaction between AE planktonic microorganisms and sampling day, illustrating distinct growth patterns depending on their presence (+) or absence (-). (F) Three-way interaction of TB, AE planktonic microorganisms, and time, demonstrating how these factors jointly influence Bd quantity under different conditions. Different letters indicate statistically significant differences across groups (*p* < 0.05). Error bars represent the 95% confidence intervals of the estimated marginal means.

Figure S8

**
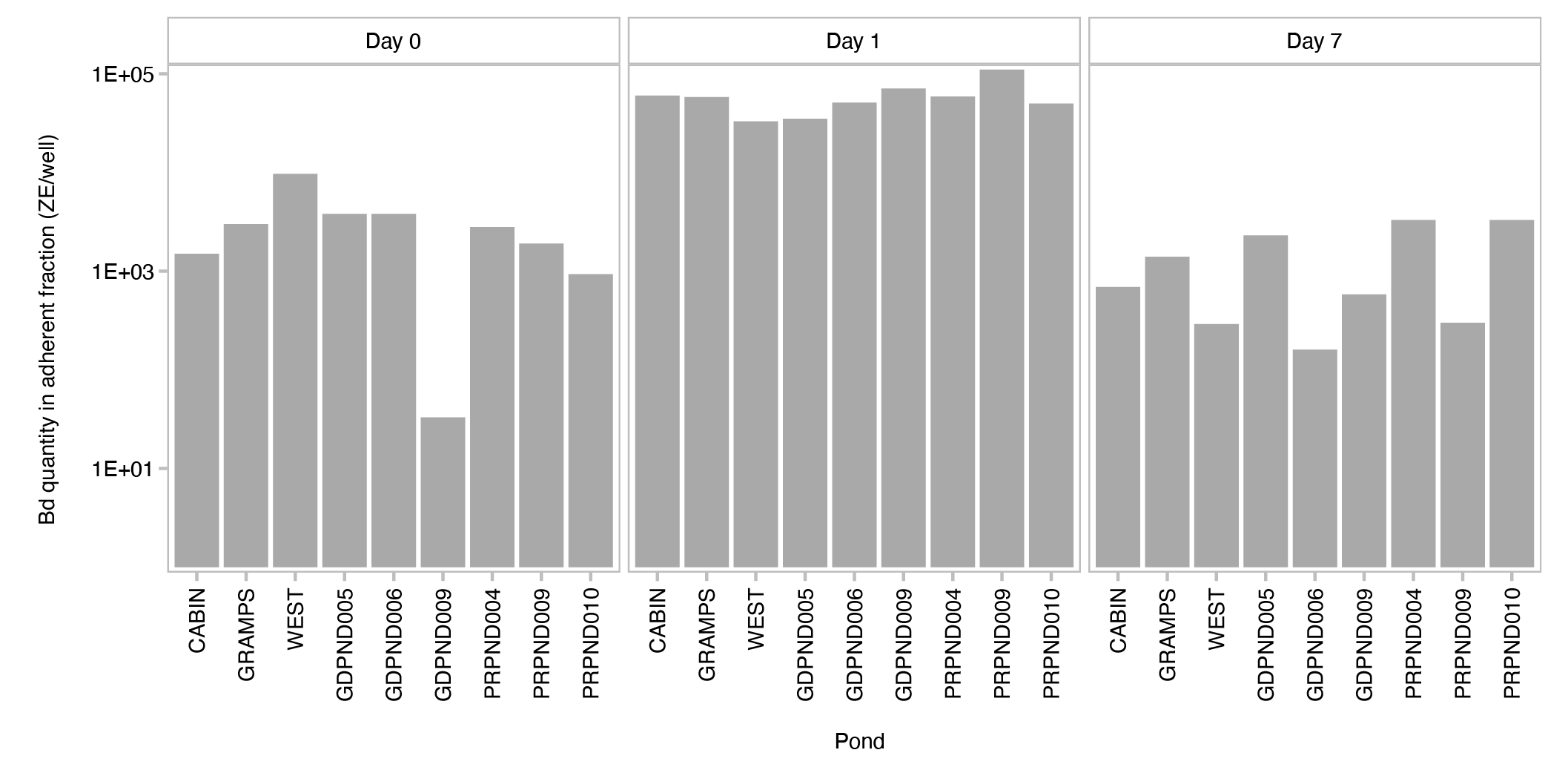
**

**Figure S8. Quantification of Bd in the presence of aquatic environmental (AE) biofilms.** Bd quantities (zoospore equivalents [ZE] per well) in adherent fractions were measured on days 0, 1, and 7 following exposures to AE biofilms collected from various ponds in the San Francisco East Bay region. Each bar represents the Bd quantity per well for a given pond sample at a given timepoint. Values were not pooled across treatments. The x-axis represents individual pond samples, and the y-axis shows Bd quantity on a logarithmic scale. The results illustrate temporal changes in Bd quantities following contact with AE biofilms.

Figure S9


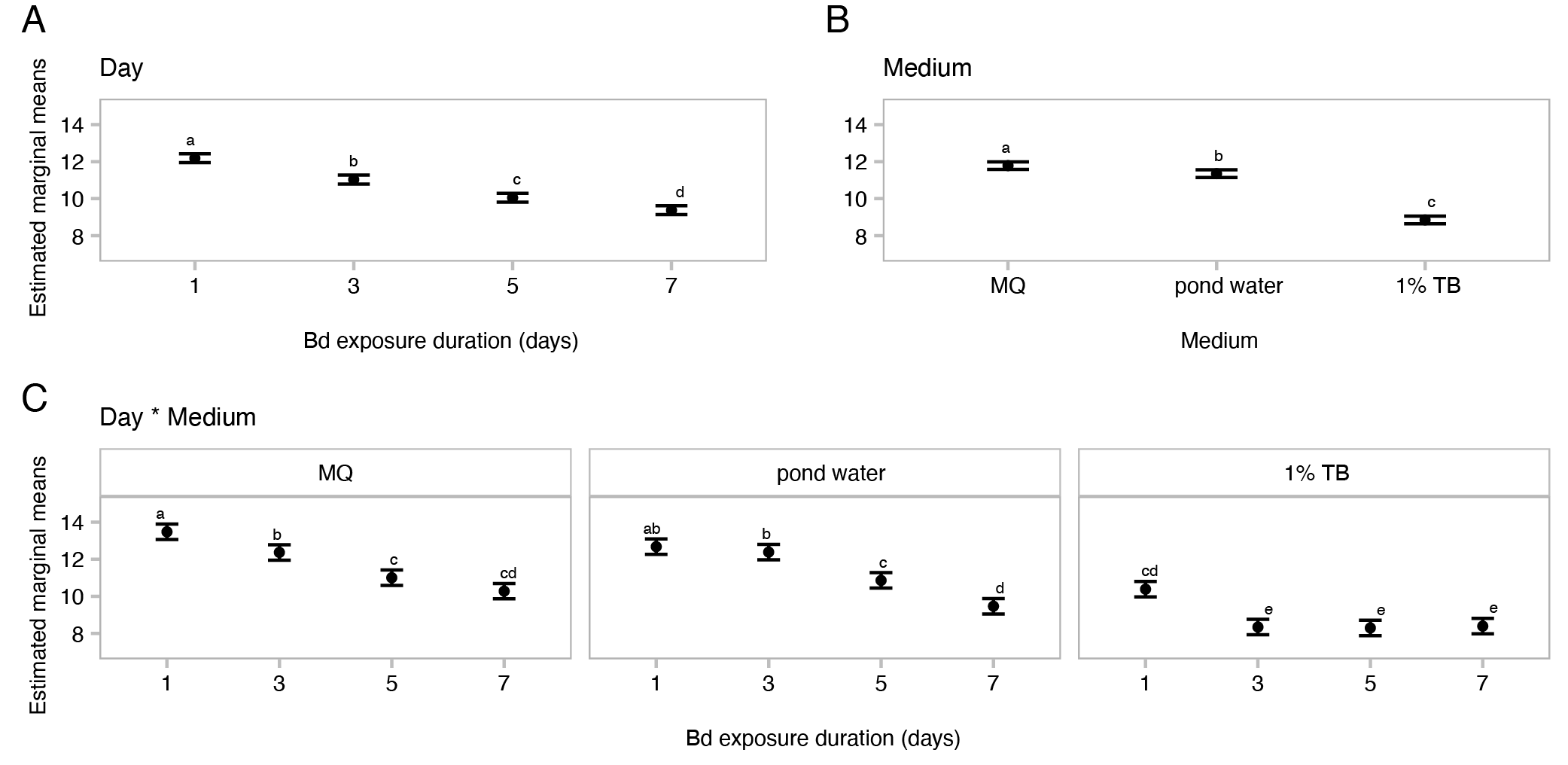


**Figure S9. Pairwise comparisons of exposure duration and medium type on Bd quantity in aquatic environmental (AE) biofilms.** Data are shown on a logarithmic y-axis. Estimated marginal means of Bd quantity (zoospore equivalents [ZE] per well) are shown across sampling days and under different media, sterile Milli-MQ (MQ) water, pond water, and pond water supplemented with 1% tryptone broth (TB). (A) Changes in Bd quantities over sampling days 1, 3, 5, and 7, with statistically significant differences indicated by distinct letters (*p* < 0.05). (B) Comparison of Bd quantity among three medium types. (C) Interaction between sampling day and medium type, showing the combined effects of exposure duration and nutrient conditions. Letters indicate statistically significant differences among groups. Error bars represent the 95% confidence intervals of the estimated marginal means.

Figure S10

**
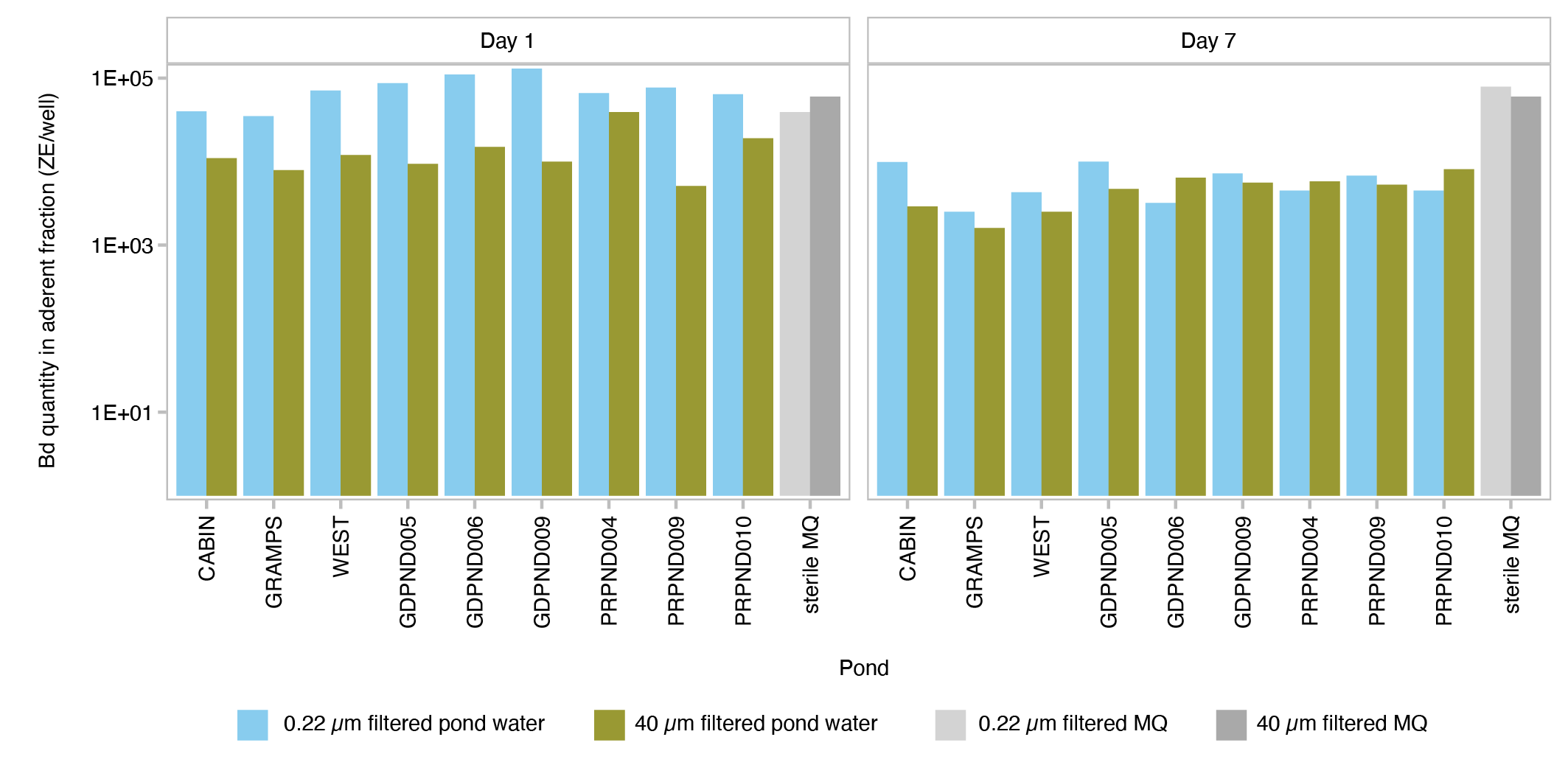
**

**Figure S10. Quantification of Bd in monolayers in the presence or absence of aquatic environmental (AE) planktonic microorganisms.** Bd quantities (zoospore equivalents [ZE] per well) in Bd monolayers were measured on days 1 and 7 following exposure to pond water collected from nine ponds in the San Francisco East Bay region. Each bar represents the Bd quantity per well for a given pond sample at a given timepoint. Values were not pooled across treatments. The x-axis represents individual pond samples, with sterile Milli-Q (MQ) water serving as control; the y-axis displays Bd quantity on a logarithmic scale. Filtration through a 40 µm filter retained AE planktonic microorganisms, whereas the 0.22 µm filter excluded them. The data illustrate differences in Bd quantity associated with filtration treatment and exposure duration.

Figure S11


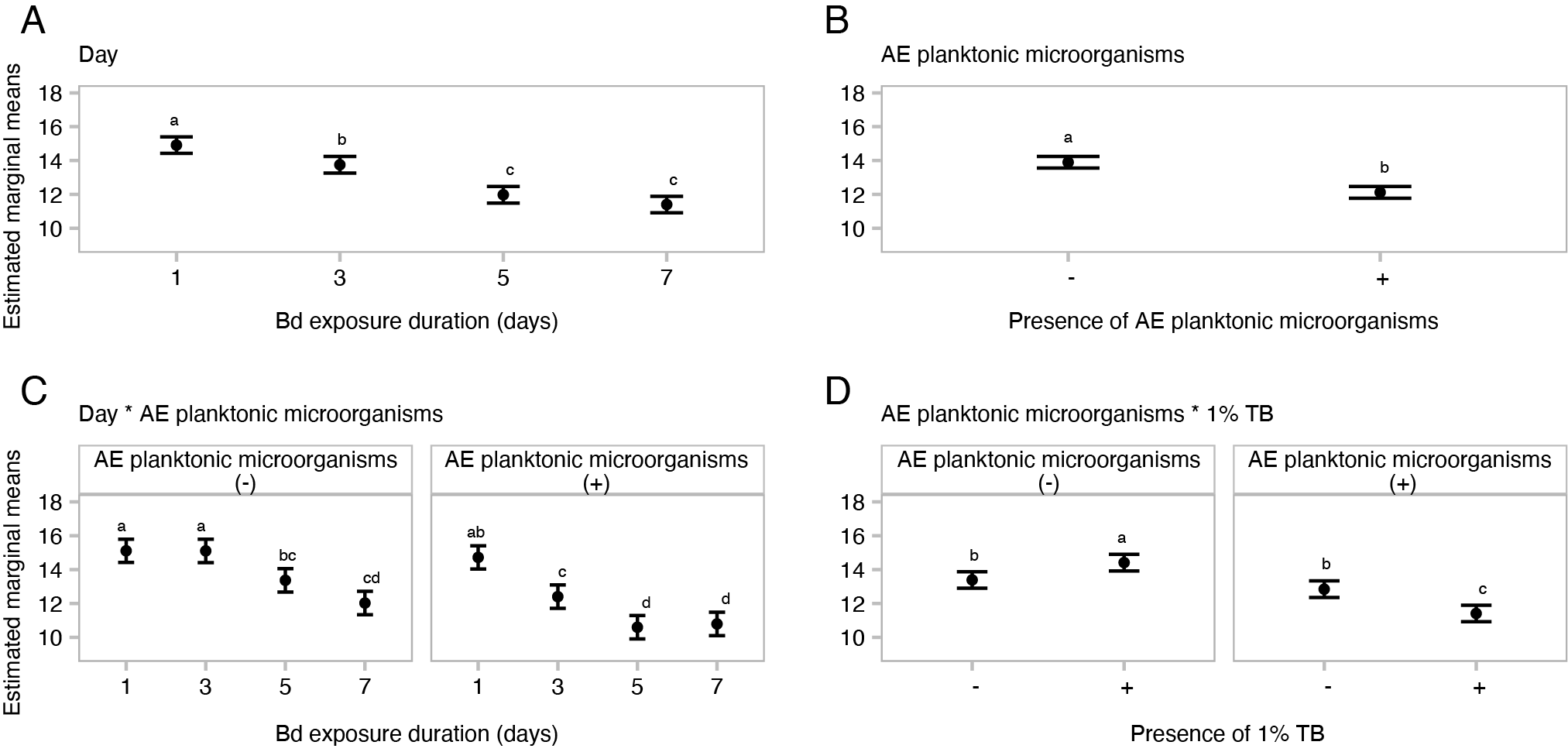


**Figure S11. Effects of exposure duration, aquatic environmental (AE) planktonic microorganisms, and 1% tryptone broth (TB) on Bd quantity in monolayers.** Data are shown on a logarithmic y-axis. (A) Temporal changes of Bd quantities over days 1, 3, 5, and 7, highlighting significant differences. (B) Influence of AE planktonic microorganisms' presence (+) or absence (-) on Bd quantity, emphasizing the role of microbial interactions. (C) Interaction between sampling day and AE planktonic microorganisms, highlighting temporal variation in microbial impact on Bd growth. (D) Combined effects of 1% TB and AE planktonic microorganisms on Bd quantity, illustrating their interaction under different microbial conditions. Letters indicate statistically significant differences within each panel (*p* < 0.05). Error bars represent 95% confidence intervals of the estimated marginal means.

End of Document
